# SIRT1 activation attenuates microglia-mediated synaptic engulfment in postoperative cognitive dysfunction

**DOI:** 10.3389/fnagi.2022.943842

**Published:** 2022-11-10

**Authors:** Yi Sun, Yuzhu Wang, Fan Ye, Victoria Cui, Dandan Lin, Hui Shi, Yan Zhang, Anshi Wu, Changwei Wei

**Affiliations:** ^1^Department of Anesthesiology, Beijing Chao-Yang Hospital, Capital Medical University, Beijing, China; ^2^Department of Anesthesiology, Xiangyang Central Hospital, Affiliated Hospital of Hubei University of Arts and Science, Xiangyang, China; ^3^Department of General Surgery, MedStar Georgetown University Hospital, Washington, DC, United States; ^4^Department of Clinical Psychology, Beijing Chao-Yang Hospital, Capital Medical University, Beijing, China; ^5^State Key Laboratory of Membrane Biology, College of Life Sciences, Peking University, Beijing, China

**Keywords:** SIRT1, microglial activation, synaptic engulfment, neuroinflammation, postoperative cognitive dysfunction

## Abstract

**Background:**

Postoperative cognitive dysfunction (POCD) is a debilitating neurological complication in surgical patients. Current research has focused mainly on microglial activation, but less is known about the resultant neuronal synaptic changes. Recent studies have suggested that Sirtuin-1 (SIRT1) plays a critical role in several different neurological disorders *via* its involvement in microglial activation. In this study, we evaluate the effects of SIRT1 activation in a POCD mouse model.

**Materials and methods:**

Exploratory laparotomy was performed in mice aged 12–14 months under sevoflurane anesthesia to establish our animal POCD model. Transcriptional changes in the hippocampus after anesthesia and surgery were evaluated by RNA sequencing. SIRT1 expression was verified by Western Blot. Mice were treated with SIRT1 agonist SRT1720 or vehicle after surgery. Changes in microglia morphology, microglial phagocytosis, presence of dystrophic neurites, and dendritic spine density were evaluated. Cognitive performance was evaluated using the Y maze and Morris water maze (MWM).

**Results:**

Sirtuin-1 expression levels were downregulated in POCD. Exposure to anesthesia and surgery lead to alteration in microglia morphology, increased synaptic engulfment, dendritic spine loss, and cognitive deficits. These effects were alleviated by SRT1720 administration.

**Conclusion:**

This study suggests an important neuroprotective role for SIRT1 in POCD pathogenesis. Increasing SIRT1 function represents a promising therapeutic strategy for prevention and treatment of POCD.

## Introduction

Postoperative cognitive dysfunction (POCD) is a frequent complication in adult surgical patients which is characterized by memory decline, inattention, and impaired orientation (Evered et al., 2018). The clinical symptoms can last for months or even years after surgery, and is associated with delayed postoperative recovery, prolonged hospitalization, and increased mortality (Skvarc et al., 2018). While neuroinflammation, increased oxidative stress, deposition of amyloid-beta, and hyperphosphorylation of tau protein have all been implicated in its development (Rosczyk et al., 2008; Skvarc et al., 2018), the overall mechanism of POCD pathogenesis remains largely obscure.

Recent research on reducing neuroinflammation in POCD has focused on microglial activation (Vizcaychipi et al., 2014; Wang H. L. et al., 2016; Li et al., 2017). Microglia originate in the yolk sac and migrate to the brain during the embryonic stage (Swinnen et al., 2013). Microglia act as the tissue-resident macrophages of the central nervous system (CNS) and are isolated from the rest of body by the blood brain barrier (Marquez-Ropero et al., 2020). During brain development, homeostatic microglia are key regulators of synaptic pruning and synaptic refinement (Paolicelli et al., 2011). In the mature brain, microglia play a surveillance role in eliminating apoptotic debris and invading pathogens (Salter and Stevens, 2017). Abnormal microglial activation contributes to diminished synaptic connectivity and function in several neurological disorders (Cardozo et al., 2019). In previous research published by our group, hippocampal microglial activation and synapse loss was observed in a POCD mouse model (Xiong et al., 2018).

Sirtuin-1 (SIRT1) is a nicotinamide adenine dinucleotide (NAD^+^)-dependent class III histone deacetylase of the sirtuin family found in a wide range of tissues including the brain (Fujita and Yamashita, 2018). SIRT1 modulates a variety of cellular processes by binding to and deacetylating various targets including nuclear factor-kappa B (NF-κB), p65, forkhead box O (FOXO), peroxisome proliferator-activated receptor alpha (PPARα), and PPAR-gamma co-activator 1-alpha (PGC-1α) (Donmez and Outeiro, 2013). SIRT1 activity is recognized to have neuroprotective properties in neurodegenerative diseases and psychiatric disorders (Gan and Tang, 2010; Abe-Higuchi et al., 2016), but little is known about its role in the development of POCD.

In this study, we demonstrate that anesthesia and surgery induce SIRT1 dysfunction and microglial activation in the hippocampus of a POCD mouse model. Administration of SIRT1 agonist SRT1720 is shown to protect against synaptic engulfment by microglia. Our data provides evidence that abnormal microglial activation mediates synaptic engulfment in POCD. SIRT1 activation may become a promising therapeutic target to prevent synapse loss and preserve cognitive function in POCD.

## Materials and methods

### Animals

Wild-type C57BL/6 mice (12–14 month of age) were used for all experiments. Mice were purchased from Vital River Laboratory Animal Technologies (Beijing, China) at 9 months old. All mice were housed on a 12-h light/dark cycle with *ad libitum* access to water and food. All experimental procedures were approved by the Institutional Animal Care and Use Committee at Capital Medical University (protocol AEEI-2020-117).

### Experimental design and animal euthanization

**FIGURE 1:**
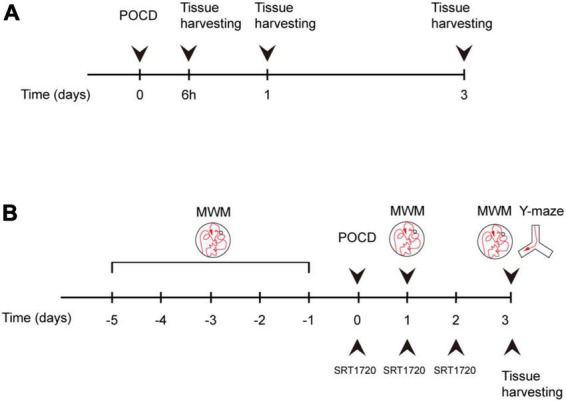
Experiment design. **(A)** Mice were assigned to control and surgery groups. Tissue was harvested at 6 h after surgery, postoperative day (POD)1, and POD3. **(B)** Surgery group mice were treated with either SRT1720 or vehicle, referred to as surgery-SRT1720 and surgery-vehicle. Tissue was harvested at POD3. Training trials of Morris water maze (MWM) were performed five consecutive days before surgery. Probe trials were performed POD1 and POD3. Y maze trials were conducted on POD3.

Animals were euthanized and tissue were harvested following behavioral tests according to experimental design (Figure 1). Overdosing inhalation anesthetic (7% sevoflurane) were administrated for euthanization as described previously (Jasien et al., 2014).

### Establishment of the postoperative cognitive dysfunction mouse model

To create our POCD mouse model, mice in the surgery group underwent exploratory laparotomy as previously described (Pan et al., 2016). Anesthesia induction was performed using 5% sevoflurane for 3 min in a closed chamber. Following induction, we moved mice out of the chamber and performed the surgical procedure under 2% sevoflurane. A midline abdominal incision of approximately 3 cm was made to expose the surgical field. The liver, stomach, spleen, kidney, bowel, and bladder were explored in sequence. The muscle, fascia, and skin were closed with 5–0 nylon sutures. Each procedure lasted around 15 min. A total of 0.2 mL of 0.2% ropivacaine was injected subcutaneously along the incision for postoperative analgesia. After the operation, sevoflurane anesthesia was terminated and the mice were returned to their cages. Body temperature was maintained at 37°C during surgery using a heating pad. Mice in the control group did not undergo any anesthesia or surgery.

### RNA-seq and bioinformatic analysis

Total RNA was extracted from the mouse hippocampus using Trizol (Tiangen, Beijing, China). Complementary DNA was synthesized with DNA polymerase I and RNaseH. Products were purified by polymerase chain reaction to generate the sequencing library and then quantified using the Agilent 2100 Bioanalyzer (Agilent Technologies, Santa Clara, CA, USA). Sequencing was performed using the Illumina HiSeq sequencer (Illumina, San Diego, CA, USA).

Data was aligned using the HISAT2 program (Johns Hopkins University, Baltimore, MD, USA) with default parameters (Liu et al., 2015). The Python package HTseq (version 5.4.2) was used for quantification and R (version 5.2.0) was used for bioinformatics analysis. DESeq2 (version 1.30.0) was used for differential expression analysis. We generated differentially expressed gene list with a *P*-value of < 0.01 and a fold change cutoff of 2. ClusterProfiler (version 3.10) was used for Gene Ontology (GO) and network analysis. We performed multiple testing correction using the Bonferroni method for genes and GO terms. Gene set enrichment analysis (GSEA) was performed using MSigDB (version 7.0).

### Drug administration

SRT1720 (S1129, Selleck, Houston, TX, USA), a SIRT1 agonist, was dissolved in dimethyl sulfoxide (DMSO) at a concentration of 40 mg/ml and stored at −20°C. SRT1720 was further diluted by saline solution during use. Mice which received SRT1720 were injected intraperitoneally (IP) at doses of 20 mg/kg immediately following exploratory laparotomy and then once daily for subsequent 2 days. The selected dose was based on previous research (Li et al., 2019). Vehicle-treated mice received 5% DMSO in saline solution.

### Western blot

Hippocampal tissues were dissected on ice. Dissected tissues were then lysed on ice for 30 min in RIPA lysis buffer (Beyotime, Jiangsu, China) with protease and phosphatase inhibitors (Roche, Manheim, Germany). Protein concentration was quantified using bicinchoninic acid (BCA) protein assay (Thermo Fisher Scientific, Waltham, MA, USA). Protein samples were run on SDS-PAGE gels and then transferred to polyvinylidene fluoride membranes (Millipore, Billerica, MA, USA). Membranes were blocked with 5% skim milk (Cell Signaling Technology, Beverly, MA, USA) for 1 h at room temperature, and subsequently incubated with mouse anti-SIRT1 (#8469, 1:1000, Cell Signaling Technology, Beverly, MA, USA) at 4°C overnight. DyLight 488 Secondary Antibodies (E032210) were used (EarthOx, San Francisco, CA, USA). ChemiDoc MP Imaging System (Bio-Rad, Hercules, CA, USA) was used to detect and quantify target bands.

### Immunohistochemistry

Mice were anesthetized with sevoflurane and then sacrificed using infusion of saline solution followed by 4% paraformaldehyde (PFA). Harvested brains were post-fixed in 4% PFA for 24 h, then cryoprotected in 30% sucrose for 48 h at 4°C. Coronal brain sections (30-μM thickness) were cut using a cryostat microtome. Brain sections were blocked with 5% bovine serum albumin for 1 h at room temperature, then incubated with goat anti-ionized calcium binding adapter molecule 1 (Iba1) (#ab5076, 1:200, abcam, Cambridge, MA, USA), rabbit anti-glia fibrillary acidic protein (GFAP) (#ab7260, 1:1000, abcam, Cambridge, MA, USA), rabbit anti-Synaptophysin (#A6344, 1:100, abclonal, Wuhan, China), and rabbit anti-lysosomal associated membrane protein 1 (LAMP1) (#ab24170, 1:200, abcam, Cambridge, MA, USA) at 4°C overnight. After washing, brain sections were incubated with fluorophore-conjugated secondary antibodies (1:500, abcam, Cambridge, MA, USA) for 1 h at room temperature. Images were visualized using the Leica TCS SP8 STED 3× confocal microscope (Leica, Wetzlar, Germany). Laser power and gain were consistent across different experiments. Quantitative analyses were performed using Fiji software.

### Three-dimensional reconstruction of microglia and engulfment analysis

Images were acquired under identical settings across all experimental groups. Brain sections were imaged with the 100× oil immersion objective (NA1.4) using the Leica TCS SP8 STED 3× confocal microscope (Leica, Wetzlar, Germany) with the 0.2 mm step. Deconvolution was performed using Huygens Professional software (SVI, Scientific Volume Imaging, Hilversum, The Netherlands). To quantify microglial engulfment of synaptophysin, images were processed using Imaris software to create a 3-dimensional (3D) surface rendering of microglia and synaptophysin location (version 9.5.0, Bitplane, Switzerland) based on the protocol from Schafer et al. (2014). Synaptophysin embedded in Iba1-positive structures were considered to be engulfed by microglia.

### Golgi staining

Hippocampal dendrites were visualized using the Golgi-Cox staining kit (#HTKNS1125, Hitobiotec, Kingsport, TN, USA). Mouse brains were harvested and immersed in impregnation solution for 2 weeks at room temperature, and then transferred to staining solution for 3 days at 4°C in accordance with manufacturer instructions. Brains were then sectioned at a thickness of 150 μM using a cryostat microtome, mounted on gelatinized slides, and stained. Images were observed with the 100× oil immersion objective (NA1.4) using the Leica TCS SP8 STED 3× confocal microscope (Leica, Wetzlar, Germany). Secondary and tertiary dendrites were sampled for spine density quantification. Counting was conducted by two experimenters independently.

### Morris water maze

The Morris water maze (MWM) has been used widely for assessment of spatial learning and memory (Vorhees and Williams, 2006). A circular pool of 120 cm diameter was filled to a depth of 30 cm with water at 23 ± 2°C. The pool was divided into four quadrants. A platform was placed 1 cm underwater in one of these quadrants and designated the target quadrant. Non-toxic titanium dioxide powder was mixed into the water to ensure the platform was not visible. Mice were allowed to swim freely for 60 s without the platform in place to promote familiarity with testing conditions. In the training trials, mice were placed in the pool facing toward the wall at one of release points. Mice were given 60 s to find the hidden platform. Mice were guided to the platform if they did not find it after 60 s. When mice reached the platform, they were allowed to stay there for 10 s. Training trials were repeated four times per day for five consecutive days before surgery, with different release points for each trial. Probe trials were conducted on postoperative day (POD) 1, 3, and 7. With the platform removed, mice were released in the opposite quadrant of the target quadrant and allowed to swim for 60 s. The escape latency, percentage of time spent in the target quadrant, and crossing platform times were analyzed using video-tracking software (ZS Dichuang Technology, Beijing, China).

### Y maze

Spontaneous alternation behavior in the Y maze was recorded to assess working memory (Lalonde, 2002). The apparatus consisted of three arms angled 120^°^ to each other. Mice were placed at the end of one arm and allowed to explore all three arms freely for 5 min. Arms were labeled A, B, and C, and the sequence of arm entries were recorded. Successful alternation was defined as the exploration of all three arms in sequence. The percent alternation was calculated based on the following equation: % alternation = [Number of successful alternations/(Total arm entries-2)] × 100 (Rosenzweig et al., 2019).

### Statistical analysis

Statistical analysis was performed with GraphPad Prism (version 7). Comparisons between two groups were performed using the Unpaired Student’s *t*-test. One-way analysis of variance (ANOVA) was used for comparisons between multiple groups. Statistical significance was set at *p* < 0.05.

## Results

### Anesthesia and surgery stimulate changes in hippocampal gene expression in mice

**FIGURE 2:**
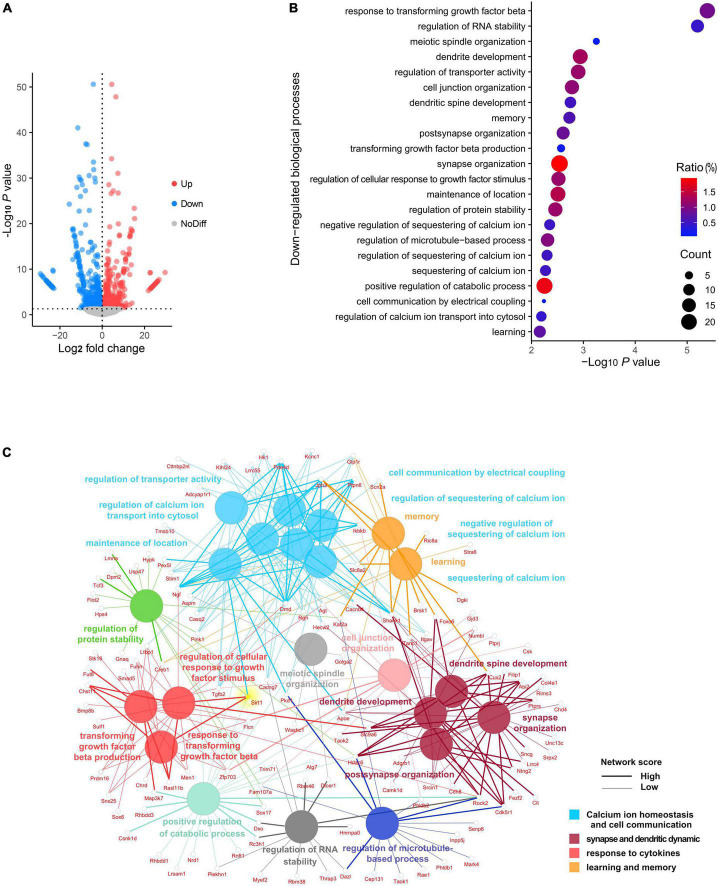
Panorama of RNA changes when compared postoperative cognitive dysfunction (POCD) to control group. **(A)** Volcano plot representation of differentially expressed gene distribution (*p* < 0.05). **(B)** Dot plot of top 22 Gene Ontology (GO) terms for downregulated biological processes. **(C)** Network analysis of top 22 downregulated biological processes among the gene interaction networks.

To investigate the landscape of gene expression after anesthesia and surgery, we performed RNA sequencing of the hippocampus from both surgery and control group mice at 72 h following exploratory laparotomy. A total of 29,037 genes were identified, of which 844 genes were significantly downregulated and 714 genes were significantly upregulated in the surgery group compared to control (Figure 2A). GO analysis was performed for significantly downregulated genes. Interestingly, the top 22 significant GO terms were enriched in four major biological processes: (i) calcium ion homeostasis and cell communication, (ii) synapse and dendrite dynamics, (iii) response to cytokines, and (iv) learning and memory (Figure 2B). We next conducted a network analysis of different GO. SIRT1, an important deacetylase, was found not only to have a high network score with the most significant GO term (response to transforming growth factor beta), but also was identified as one of the key hubs connecting several significant biological processes (Figure 2C). These results suggested SIRT1 acts as a crucial regulator of biological processes in the hippocampus following anesthesia and surgery.

### Anesthesia and surgery induce SIRT1 downregulation and microglial activation in the hippocampus

**FIGURE 3:**
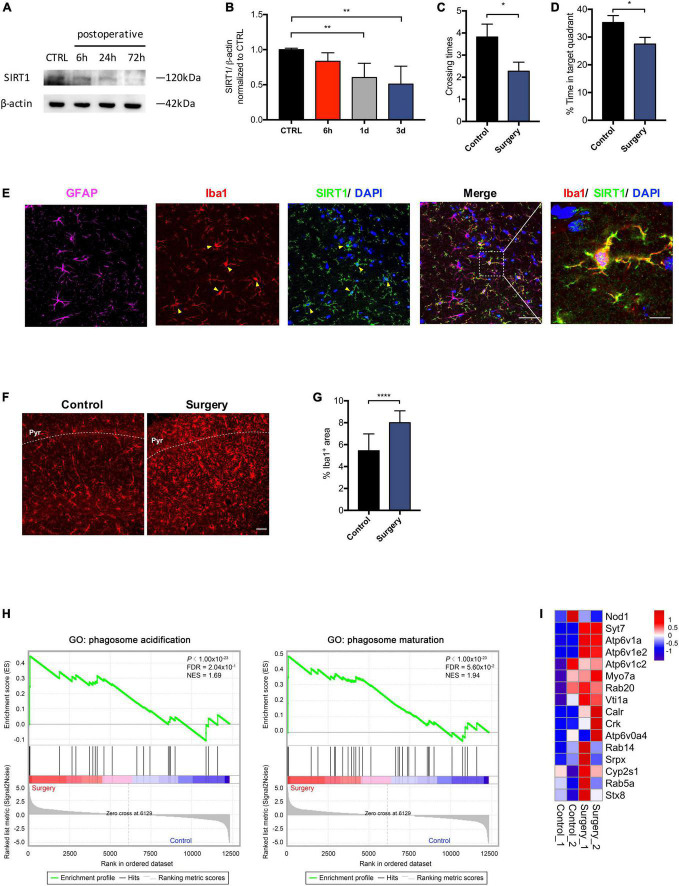
Anesthesia and surgery induces microglial activation in the hippocampus. **(A)** Time course of SIRT1 expression. **(B)** Qualification of panel **A**. One-way analysis of variance (ANOVA) with Dunnett *post-hoc* test. Mean ± SD. **(C)** Crossing times of the platform area in probe trials on the third after surgery. *n* = 11 per group. Two sided *t*-test. Mean ± SEM. **(D)** Percentage of time spent in target quadrant in probe trials on the third after surgery. *n* = 11 per group. Two sided *t*-test. Mean ± SEM. **(E)** Representative images of SIRT1 co-labeled with antibodies to cell-type-specific markers, *n* = 5. Left panel scale bar, 50 μm; right panel scale bar, 10 μm. **(F)** Representative images of Iba1 (Red) labeling in the CA1. Scale bar, 50 μm. Pyr, pyramidal cell layer. **(G)** Qualification of panel **F**, *n* = 5 per group. Two sided *t*-test. Mean ± SD. **(H)** Gene set enrichment analyses (GSEA) of phagosome acidification (left) and phagosome maturation (right) in surgery versus control group. NES, normalized enrichment score; FDR, false discovery rate. **(I)** Heatmap representation of differential expression for phagosome acidification and phagosome maturation genes. *p < 0.05; **p < 0.01; ****p < 0.0001.

Microglia are responsive to SIRT1 signaling (Merlo et al., 2020). We sought to investigate the impact of anesthesia and surgery on microglia in the hippocampus. We examined levels of SIRT1 in the hippocampus at 6, 24, and 72 h after anesthesia and surgery. Compared to control, SIRT1 expression in the surgery group declined significantly at 24 and 72 h postoperatively (*p* < 0.01; Figures 3A,B). We performed probe trial of MWM at 72 h postoperatively. Anesthesia and surgery resulted in significant decreased crossing times of the platform (control vs. surgery, *p* = 0.0423; Figure 3C) and percentage of time spent in target quadrant (control vs. surgery, *p* = 0.0373; Figure 3D). To elucidate the modulatory mechanisms of SIRT1 in POCD, we then identified cellular targets of SIRT1 by immunohistochemistry. We found that SIRT1 co-localized with microglia marker Iba1, but not astrocyte marker GFAP (Figure 3E). In the surgery group, immunohistochemistry revealed significantly increased expression of microglia marker Iba1 in the hippocampal CA1 region (control vs. surgery *p* < 0.0001; Figures 3F,G). GSEA of our RNA sequencing also identified upregulation of the biological processes phagosome acidification and phagosome maturation in the surgery group compared to control (Figures 3H,I).

### SIRT1 activation prevents alteration in microglia morphology after anesthesia and surgery

**FIGURE 4:**
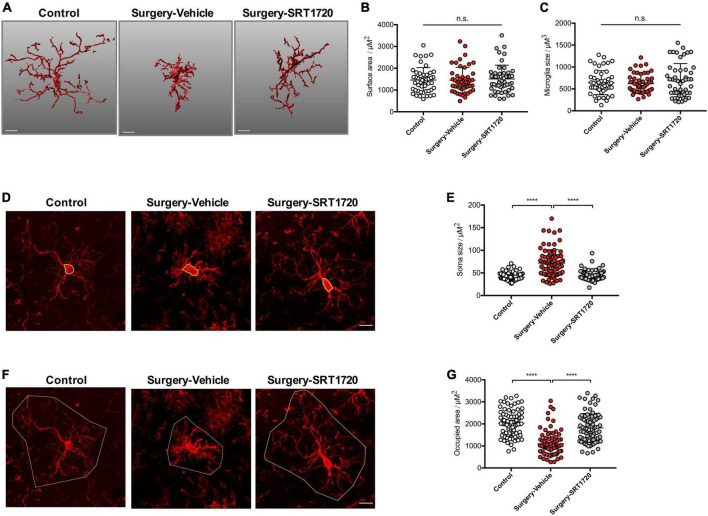
SIRT1 activation prevents alteration in microglia morphology after anesthesia and surgery. **(A)** Representative 3D reconstruction and rendering of microglia. Scale bar, 10 μm. **(B)** Quantification of surface area of microglia, *n* = 5 per group; 10 cells per mouse quantified. **(C)** Quantification of microglia size, *n* = 5 per group; 10 cells per mouse quantified. **(D)** Representative images of microglia immunohistochemistry (Iba1, red), with measurement of soma size (yellow outline). Scale bar, 10 μm. **(E)** Quantification of microglial soma size, *n* = 5 per group; 14–18 cells per mouse quantified. **(F)** Representative images of microglia immunohistochemistry, with measurement of occupied area (yellow outline). Scale bar, 10 μm. **(G)** Quantification of microglial occupied area, *n* = 5 per group; 14–18 cells per mouse quantified. One-way analysis of variance (ANOVA) with Tukey *post-hoc* test. All data are shown as mean ± SD. n.s., not significant; ****p < 0.0001.

Microglial activation is associated with specific changes in cell morphology (Torres-Platas et al., 2014). To investigate whether SIRT1 activation affects hippocampal microglial activation after anesthesia and surgery, we administrated intraperitoneal SRT1720, an agonist of SIRT1, to mice in the surgery group. Quantitative morphometric 3D measurements of microglia identified no significant difference in surface area (*p* = 0.8811; Figures 4A,B) and microglia size (*p* = 0.3919; Figures 4A,C) among the control, surgery-vehicle, and surgery-SIRT1720 groups. We further calculated microglia soma size and area of the hippocampus occupied by microglia. Compared with control, the surgery group exhibited increased soma size in hippocampal microglia (control vs. surgery-vehicle, *p* < 0.0001; Figures 4D,E), and decreased occupied area (control vs. surgery-vehicle, *p* < 0.0001; Figures 4F,G). These alterations in microglia morphology were reversed by administration of SIRT1 agonist, with decreased soma size in the surgery-SIRT1720 group compared to the surgery-vehicle group (*p* < 0.0001; Figures 4D,E) and increased occupied area (surgery-vehicle vs. surgery-SIRT1720, *p* < 0.0001; Figures 4F,G).

### SIRT1 activation prevents microglia-mediated synaptic engulfment after anesthesia and surgery

**FIGURE 5:**
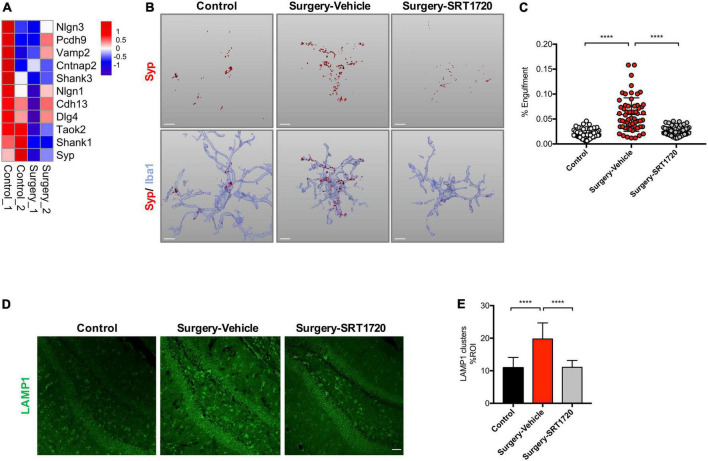
SIRT1 overexpression ameliorates microglia engulfment of presynaptic inputs after surgery. **(A)** Heatmap of synaptic protein related gene expression. **(B)** Representative 3D reconstruction and rendering of Syp signals inside Iba1 + microglia from each experimental group. Scale bar, 5 μm. **(C)** Quantification of panel **B**, *n* = 5 per group; 10–15 cells per mouse were quantified. **(D)** Maximum *z* projections showing LAMP1 in the dentate gyrus of hippocampus from each experimental group. Scale bar, 50 μm. **(E)** Quantification of panel **D**, *n* = 5 per group. One-way analysis of variance (ANOVA) with Tukey *post-hoc* test. Data are shown as mean ± SD. ****p < 0.0001. Syp, synaptophysin.

Microglia regulate synaptic connections *via* synaptic engulfment and elimination, processes which have been implicated in memory degradation and forgetting (Wang et al., 2020). Interestingly, genes related to synaptic proteins were downregulated in the hippocampus of the surgery group (Figure 5A). We evaluated synapse engulfment by measuring the presynaptic marker synaptophysin in microglia. Three-dimensional surface reconstruction and rendering demonstrated that the volume of synaptophysin within microglia increased significantly in mice after anesthesia and surgery (control vs. surgery-vehicle, *p* < 0.0001; Figures 5B,C). This effect was eliminated by SIRT1 activation *via* its agonist SIRT1720 (surgery-vehicle vs. surgery-SIRT1720, *p* < 0.0001; Figures 5B,C).

The lysosomal marker LAMP1 was found to be enriched in dystrophic neurites (Zhong et al., 2019). There was a significant elevation of LAMP1 in the hippocampal dentate gyrus in the surgery group compared to control (*p* < 0.0001; Figures 5D,E). Surgery-induced LAMP1 elevation were reversed by SIRT1 activation (surgery-vehicle vs. surgery-SRT1720, *p* < 0.0001; Figures 5D,E).

### SIRT1 activation reverses anesthesia and surgery-induced spine loss in hippocampal neurons

**FIGURE 6:**
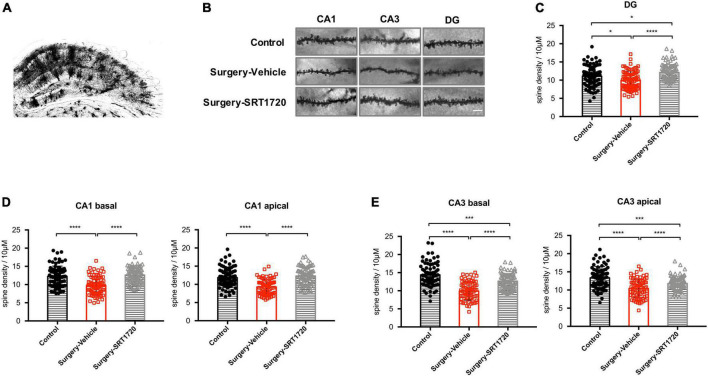
SIRT1 activation restores surgery-induced spine loss. **(A)** Representative images of Golgi staining of hippocampus. **(B)** High-magnification representative images of dendritic spine from each experimental group. Scale bar, 5 μm. **(C)** Spine density of dentate gyrus (DG) neurons. *n* = 80–84 spines from four mice per group. **(D)** Spine density in apical and basal dendrites of CA1 neurons, *n* = 78–80 spines from four mice per group. **(E)** Spine density in apical and basal dendrites of CA3 neurons. *n* = 74–80 spines from four mice per group. One-way analysis of variance (ANOVA) with Tukey *post hoc* test. All data are shown as mean ± SD. *p < 0.05; ***p < 0.001; ****p < 0.0001.

To further assess the impact of synaptic engulfment by microglia, we performed Golgi staining on the mouse hippocampus. We found that anesthesia and surgery induced significant neuronal spine loss in the CA1 region (control vs. surgery-vehicle, *p* < 0.0001; Figures 6A,B,D), which was reversed by SRT1720 treatment (surgery-vehicle vs. surgery-SRT1720, *p* < 0.0001; Figures 6A,B,D). We also identified reduced spine density in neurons of the dentate gyrus (control vs. surgery-vehicle, *p* < 0.05; Figures 6B,C) and the CA3 region (control vs. surgery-vehicle, *p* < 0.0001; Figures 6B,E) in the surgery-vehicle group. SIRT1 activation partially restored spinal loss (surgery-vehicle vs. surgery-SRT1720, *p* < 0.0001; Figures 6B,C,E).

### SIRT1 activation reduces cognitive deficits after anesthesia and surgery

**FIGURE 7:**
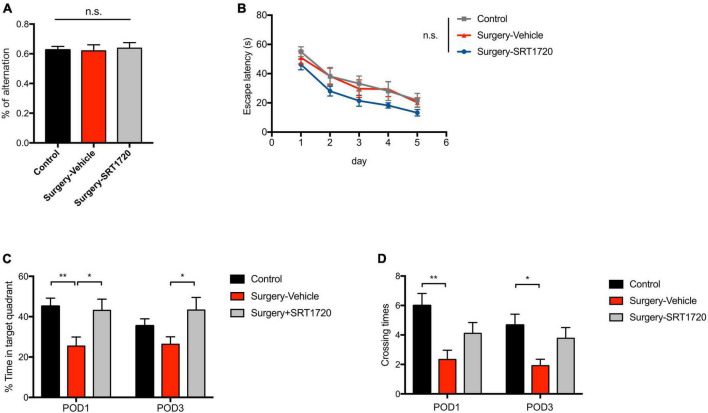
SIRT1 activation overcomes loss of cognitive performance after surgery and anesthesia. **(A)** Y-maze performance on the third day after surgery. *n* = 11 for control, *n* = 6 for surgery-vehicle, *n* = 7 for surgery-SRT1720. One-way analysis of variance (ANOVA) with Tukey *post-hoc* test. **(B)** Escape latency in training trials of Morris water maze (MWM). *n* = 12 for control and surgery-vehicle, *n* = 9 for surgery-SRT1720. Two-way ANOVA with Tukey *post hoc* test. **(C)** Percentage of time spent in target quadrant in probe trials on the first and third after surgery. *n* = 12 for control and surgery-vehicle, *n* = 9 for surgery-SRT1720. Two-way ANOVA with Tukey *post-hoc* test **(D)** Crossing times of the platform area in probe trials on the first and third after surgery. *n* = 12 for control and surgery-vehicle, *n* = 9 for surgery-SRT1720. Two-way ANOVA with Bonferroni *post hoc* test. All data are shown as mean ± SEM. n.s., not significant; *p < 0.05; **p < 0.01. POD, postoperative day.

To further examine the effects of SIRT1 activation on cognitive performance, the Y maze was used to evaluate working memory. The control and surgery-vehicle groups performed similarly in percentage of spontaneous alternation (*p* = 0.9343; Figure 7A). The MWM was used to assess spatial reference memory. Escape latency decreased over subsequent training days (*p* < 0.0001; Figure 7B), with no significant difference among groups (*p* = 0.9988; Figure 7B). Probe trials were conducted on POD1 and POD3. There was a significant reduction in percentage time spent in the target quadrant in surgery-vehicle mice on POD1 compared to control (*p* = 0.0044; Figure 7C). Surgery-vehicle group mice also displayed fewer crossing times of the platform on POD1 (control vs. surgery-vehicle, *p* = 0.0014) and POD3 (control vs. surgery-vehicle, *p* = 0.0278; Figure 7D). Treatment with SRT1720 resulted in improved percentage of time spent in target quadrant on POD1 and POD3 (surgery-vehicle vs. surgery-SRT1720, *p* < 0.05; Figure 7C).

## Discussion

Postoperative cognitive dysfunction is a common neurological complication for surgical patients, especially for the elderly (Evered et al., 2011). Microglial activation and neuroinflammation have long been thought to represent the underlying mechanisms of POCD development (Skvarc et al., 2018). Microglia serve as the innate immune cells in brain, surveilling and maintaining homeostasis in the CNS (Marquez-Ropero et al., 2020). Proliferation and activation of microglia is a key process in several neurodegenerative diseases (Hansen et al., 2018). In this study, we found that anesthesia and surgery induced SIRT1 downregulation in the hippocampus of our POCD mouse model. Abnormal microglial activation after anesthesia and surgery led to increased synaptic engulfment and spine loss, resulting in cognitive impairment, effects which were reversed by SRT1720 treatment. Our data thus suggest a potentially neuroprotective role for SIRT1 in preventing POCD.

In our POCD model, GO and network analysis identified the SIRT1 gene as significantly downregulated after anesthesia and surgery. Additionally, SIRT1 was identified as a key hub connecting genes in related biological pathways. SIRT1 is a (NAD^+^)-dependent deacetylases that modulate multiple cellular processes (Donmez and Outeiro, 2013), and has reported neuroprotective role in several neurodegenerative diseases (Gan and Tang, 2010) and psychiatric disorders (Abe-Higuchi et al., 2016). We observed decreased SIRT1 expression in the hippocampus at POD1 and POD3, which is consistent with previous studies (Yan et al., 2019, 2020). SIRT1 is enriched in the nuclei and cytoplasm of microglia, suggesting microglia as the target of SIRT1 in the hippocampus. We found that SIRT1 activation attenuates alteration in microglia morphology induced by anesthesia and surgery. The decrease in microglia-mediated engulfment of synaptic protein detected in mice rescued with SRT1720 was associated with decreased neuronal spine loss. Administration of SIRT1 agonist SRT1720 also partially reversed cognitive deficits in mice after anesthesia and surgery. The neuroprotective role of SRT1720 has previously been demonstrated in mice after cardiac surgery, with administration associated with significantly reduced plasma inflammatory cytokine levels (Shi et al., 2019).

Our findings of increased microglial activation following exposure to anesthesia and surgery is consistent with several previous studies (Hovens et al., 2013; Wang Y. et al., 2016; Zhang et al., 2017). The leakage of peripheral cytokines through the blood-brain barrier has been proposed as a trigger for microglial activation (Skvarc et al., 2018). Previous studies on animal models of other neurodegenerative disease have demonstrated specific alterations in microglia morphology with microglial activation (Werneburg et al., 2020; Xu et al., 2020). Ramified microglia are characterized by small cell bodies and long processes and are thought to perform an active surveillance role in homeostatic neural environments (Vinet et al., 2012). When activated, ramified microglia convert to the amoeboid subtype, which is characterized by expanded cell bodies and retracted processes (Yuan et al., 2020). Although no significant changes were observed in surface area and microglial size, we found decreased occupied hippocampal area and increased soma size of hippocampal microglia in our POCD mouse model.

Microglial morphologic alteration to the amoeboid subtype is strongly correlated with increased phagocytic activity (Diaz-Aparicio et al., 2016). Synapse engulfment and loss mediated by activated microglia has been demonstrated in animal models of Alzheimer’s disease (Hansen et al., 2018), epilepsy (Andoh et al., 2019), and demyelinating diseases such as multiple sclerosis (Werneburg et al., 2020). In our study, RNA sequencing and GSEA revealed increased expression of phagosome acidification and maturation genes accompanied by downregulation of synaptic protein genes in the hippocampus of the surgery group. Our group previously demonstrated a linear relationship between synaptic protein loss and CD68 immunoreactivity, which was used in that study as a marker of microglial phagocytosis (Xiong et al., 2018). In the current study, we employed 3D reconstruction and rendering of microglia to observe that microglia from surgery group mice contained a higher amount of engulfed synaptophysin compared to control. Higher levels of the lysosomal marker LAMP1, which is enriched in dystrophic neurites, were found in the hippocampal dentate gyrus of the surgery group. Together, our data suggest that microglia-mediated synaptic engulfment represents one of the key processes underlying POCD.

Excessive synaptic pruning by microglia has been implicated in schizophrenia (Sellgren et al., 2019) and is associated with the forgetting of memory (Wang et al., 2020). In our study, Golgi staining detected significantly reduced spine density in the dentate gyrus, CA1, and CA3 regions of the hippocampus in the surgery group. These findings are consistent with previous work, also in mouse models, demonstrating dendritic spine loss in POCD (Qiu et al., 2020). In behavioral testing performed after anesthesia and surgery, we did not detect deficits in working memory but did identify impairment in spatial reference memory at POD1 and POD3.

Our study has several limitations. The sample size for RNA sequencing was small. We did not directly demonstrate the engulfment of postsynaptic elements, and we also did not elucidate the signaling mechanisms by which SIRT1 overexpression reduces microglial activation. A previous study has suggested a potential link between SIRT1 activation and inhibition of nuclear factor-kappa B (NF-κB) *in vitro* (Tong et al., 2020). Further investigation is needed to address these issues.

## Conclusion

Abnormal microglial activation and resultant synaptic engulfment represent an important pathway underlying the development of POCD. In this study, reversal of SIRT1 downregulation with its agonist SRT1720 alleviated microglial activation, decreased engulfment of synaptic elements, reduced neuronal spine loss, and improved cognitive performance in our POCD mouse model. Increasing SIRT1 function is a promising therapeutic target for the prevention of POCD.

## Data availability statement

The data presented in this study are deposited in the GEO repository, accession number: GSE215410.

## Ethics statement

This animal study was reviewed and approved by the Institutional Animal Care and Use Committee at Capital Medical University.

## Author contributions

CW and YS: study design. YS, FY, and DL: performed research. YS and YW: analyzing data. YS: writing – original draft preparation. CW, YW, HS, and VC: writing – review and editing. YZ and AW: supervision. CW and AW: funding acquisition. All authors approved the final manuscript.

## Abbreviations

POCD: postoperative cognitive dysfunction
SIRT1: Sirtuin-1
NAD: nicotinamide adenine dinucleotide
NF-κ B: nuclear factor-kappa B
FOXO: forkhead box O
PPARα: peroxisome proliferator-activated receptor alpha
PGC-1α: PPAR-gamma co-activator 1-alpha
CNS: central nervous system
DMSO: dimethyl sulfoxide
BCA: bicinchoninic acid
PFA: paraformaldehyde
Iba1: ionized calcium binding adapter molecule 1
GFAP: glia fibrillary acidic protein
SYP: synaptophysin
LAMP1: lysosomal associated membrane protein 1
MWM: Morris water maze
ANOVA: one-way analysis of variance
POD: postoperative day.
