# Supplementary figures and images for: SIRT1 activation attenuates microglia-mediated synaptic engulfment in postoperative cognitive dysfunction
Source: Front Aging Neurosci. 2022 Nov 10;14:943842. doi: 10.3389/fnagi.2022.943842 (PMC9685341; doi:10.3389/fnagi.2022.943842)

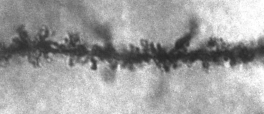

Supplement: Supplementary file 1 [file Data_Sheet_1.zip › raw data 2/Fig6/*golgi_ctrl_CA3_basal_3122_cut.tif]

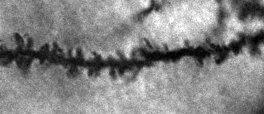

Supplement: Supplementary file 1 [file Data_Sheet_1.zip › raw data 2/Fig6/*20201024 golgi SRT.lif_SIRT1_CA1_basal_3226_ch01-1.jpg]

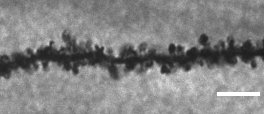

Supplement: Supplementary file 1 [file Data_Sheet_1.zip › raw data 2/Fig6/*golgi SIRT1_DG_3116_ch01-1 scale bar.jpg]

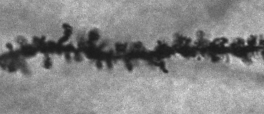

Supplement: Supplementary file 1 [file Data_Sheet_1.zip › raw data 2/Fig6/*20201203 golgi CTRL.lif_CTRL_CA1_basal_15_ch01-1.tif]

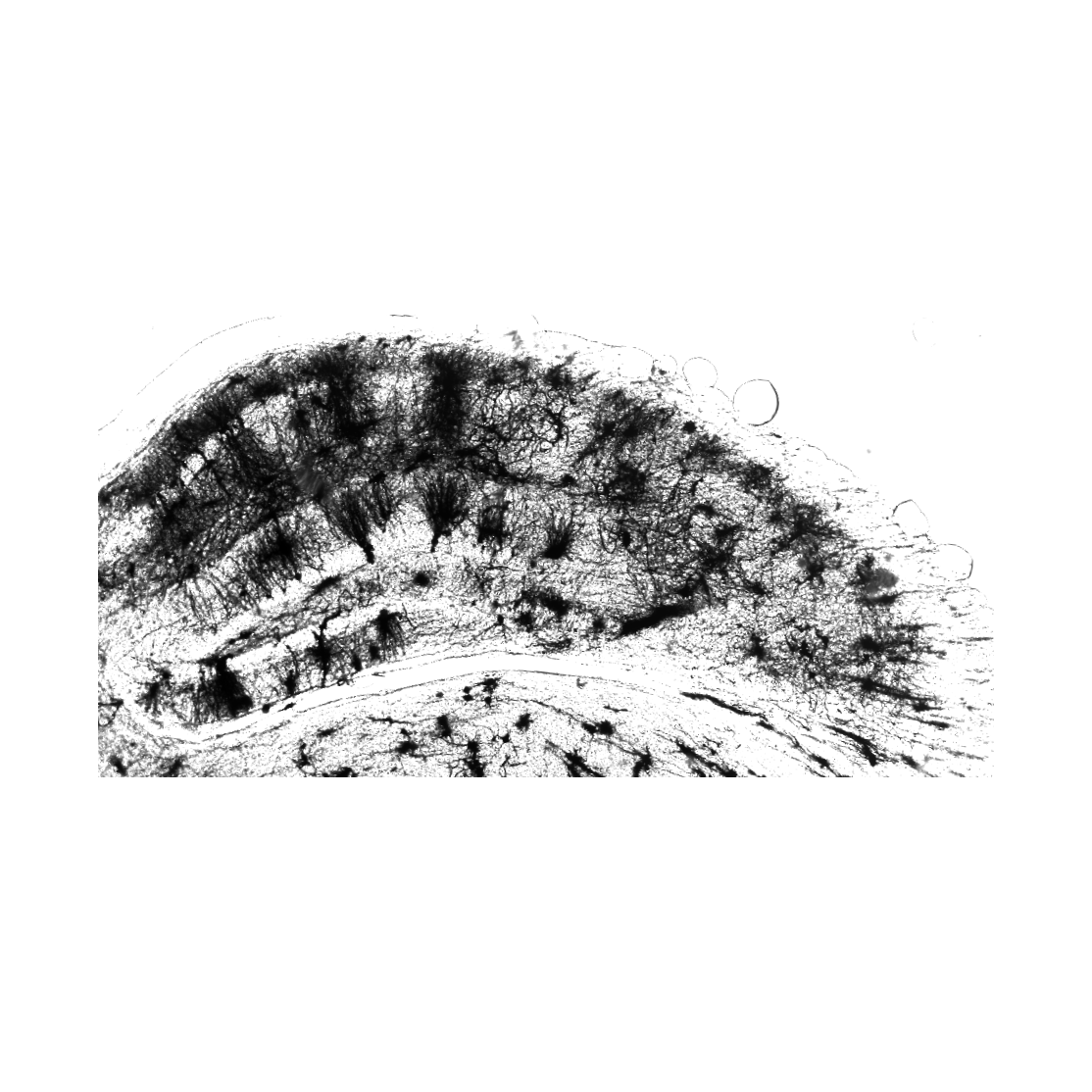

Supplement: Supplementary file 1 [file Data_Sheet_1.zip › raw data 2/Fig6/golgi dibeijing.tif]

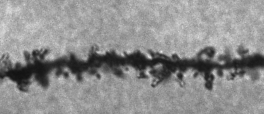

Supplement: Supplementary file 1 [file Data_Sheet_1.zip › raw data 2/Fig6/*golgi_ctrl_CA3_DG_3128_ch01-1.tif]

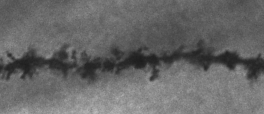

Supplement: Supplementary file 1 [file Data_Sheet_1.zip › raw data 2/Fig6/*golgi_POCD7_DG_2214_ch01-1.tif]

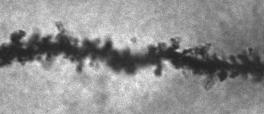

Supplement: Supplementary file 1 [file Data_Sheet_1.zip › raw data 2/Fig6/*golgi SRT.lif_SIRT1_CA3_basal_1220_cut.tif]

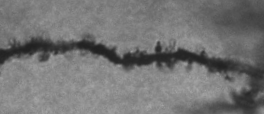

Supplement: Supplementary file 1 [file Data_Sheet_1.zip › raw data 2/Fig6/*golgi_POCD7_CA3_basal_2121_cut.tif]

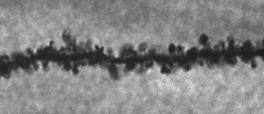

Supplement: Supplementary file 1 [file Data_Sheet_1.zip › raw data 2/Fig6/*golgi SRT.lif_SIRT1_DG_3116_ch01-1.tif]

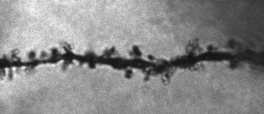

Supplement: Supplementary file 1 [file Data_Sheet_1.zip › raw data 2/Fig6/*20201205 golgi POCD.lif_POCD7_CA1_apical_01_ch01-1.tif]

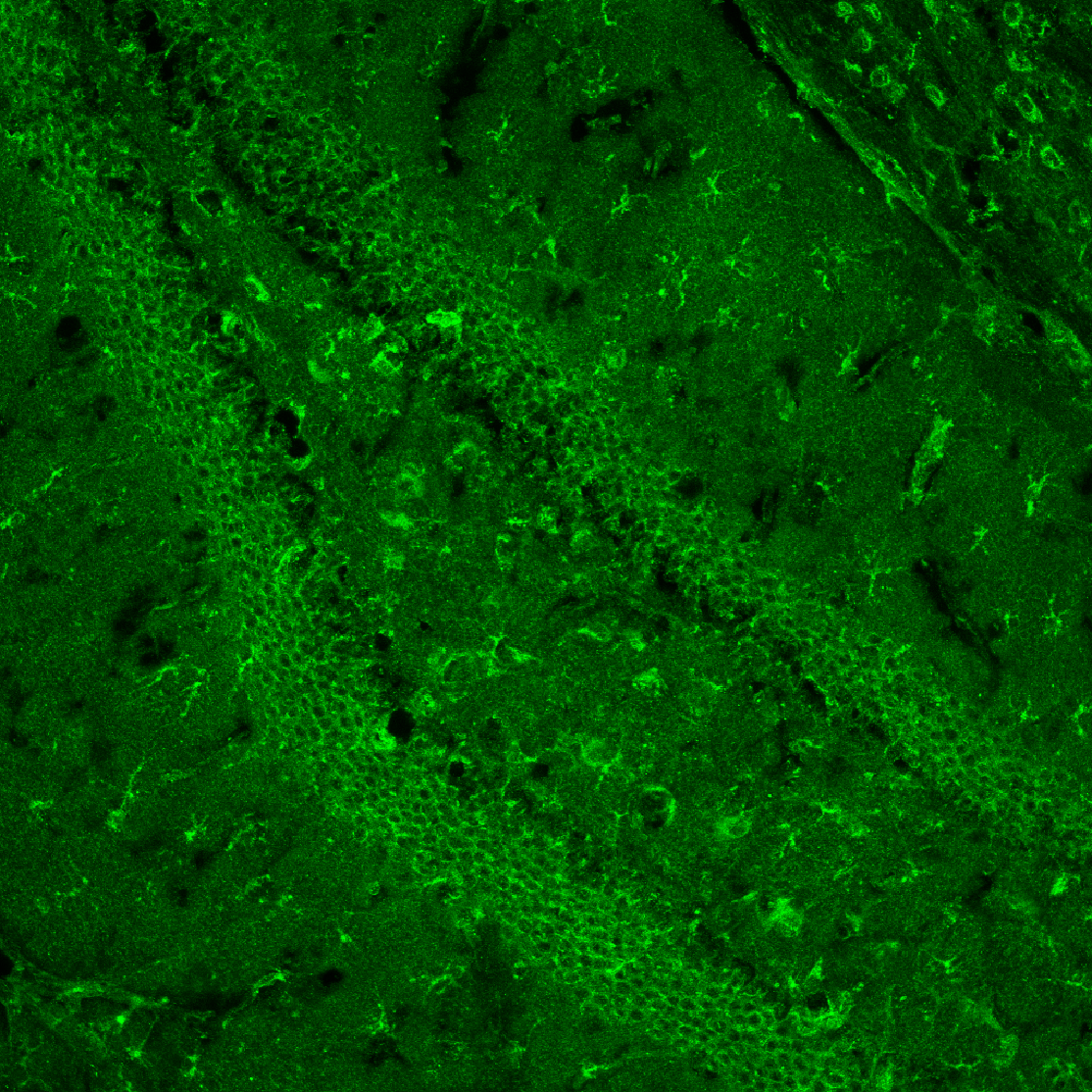

Supplement: Supplementary file 1 [file Data_Sheet_1.zip › raw data 2/FIg5/*20191017 lamp1001_POCD7_DG_LAMP1 488_1211Snapshot1_ch00.tif]

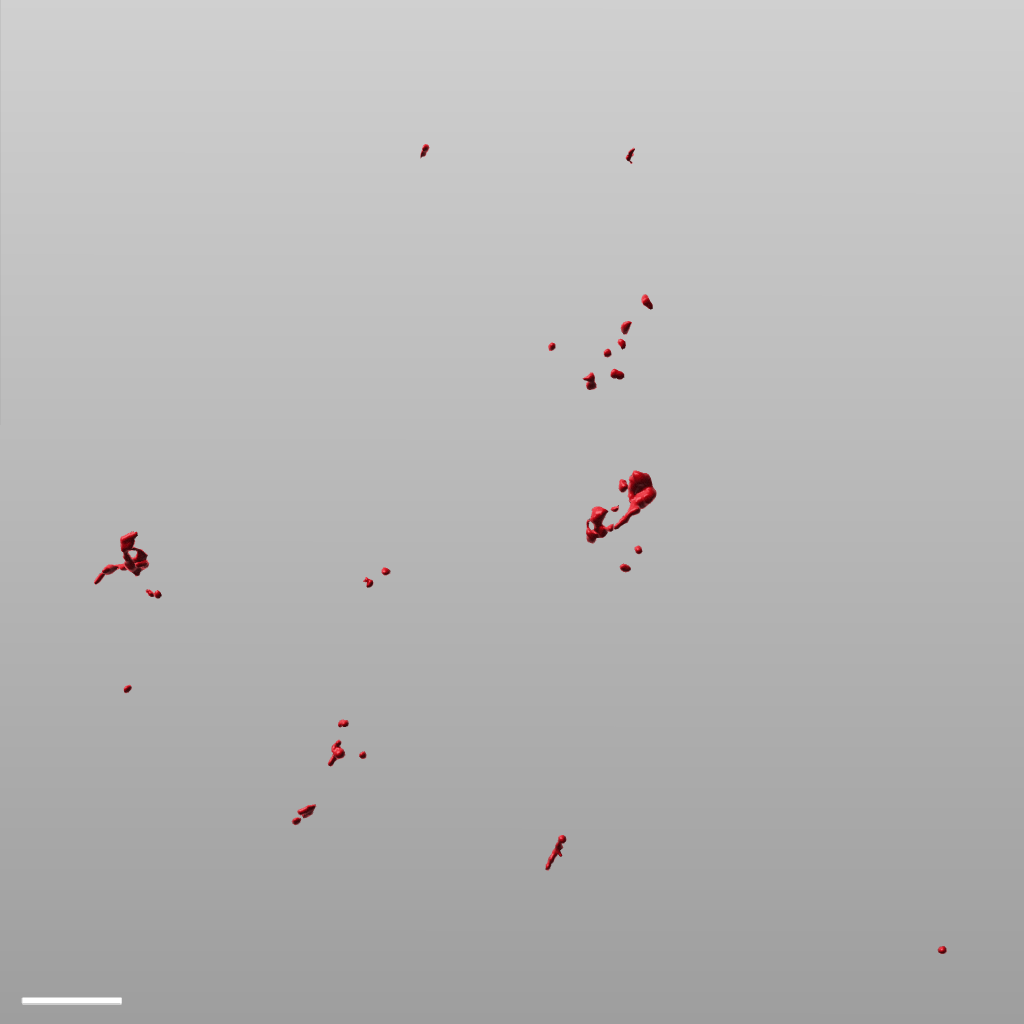

Supplement: Supplementary file 1 [file Data_Sheet_1.zip › raw data 2/FIg5/1231 iba Imported From Huygens_[ii1_CTRL_CA1a_1122_decon_converted_Image_2]_2020-11-24T19-49-36.918.tif]

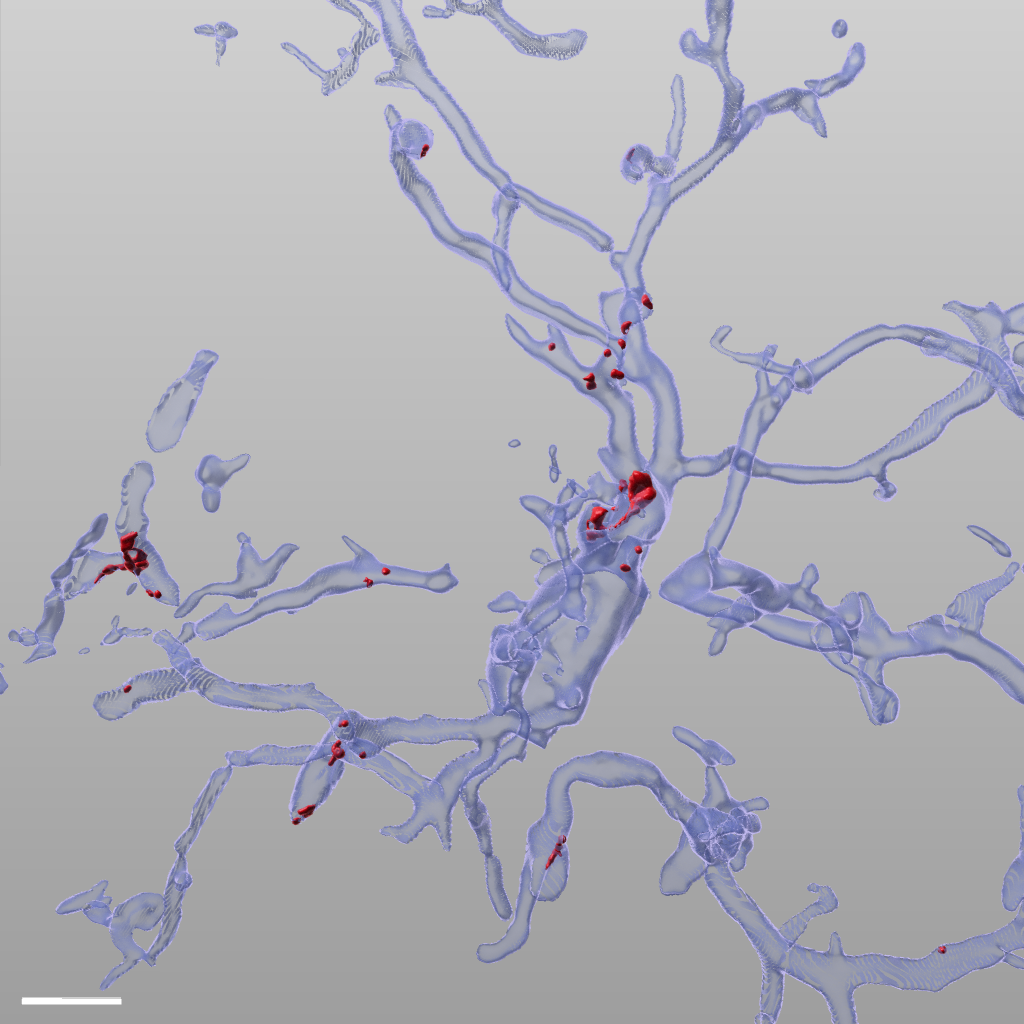

Supplement: Supplementary file 1 [file Data_Sheet_1.zip › raw data 2/FIg5/1231 iba Imported From Huygens_[ii1_CTRL_CA1a_1122_decon_converted_Image_2]_2020-11-24T19-52-39.333.tif]

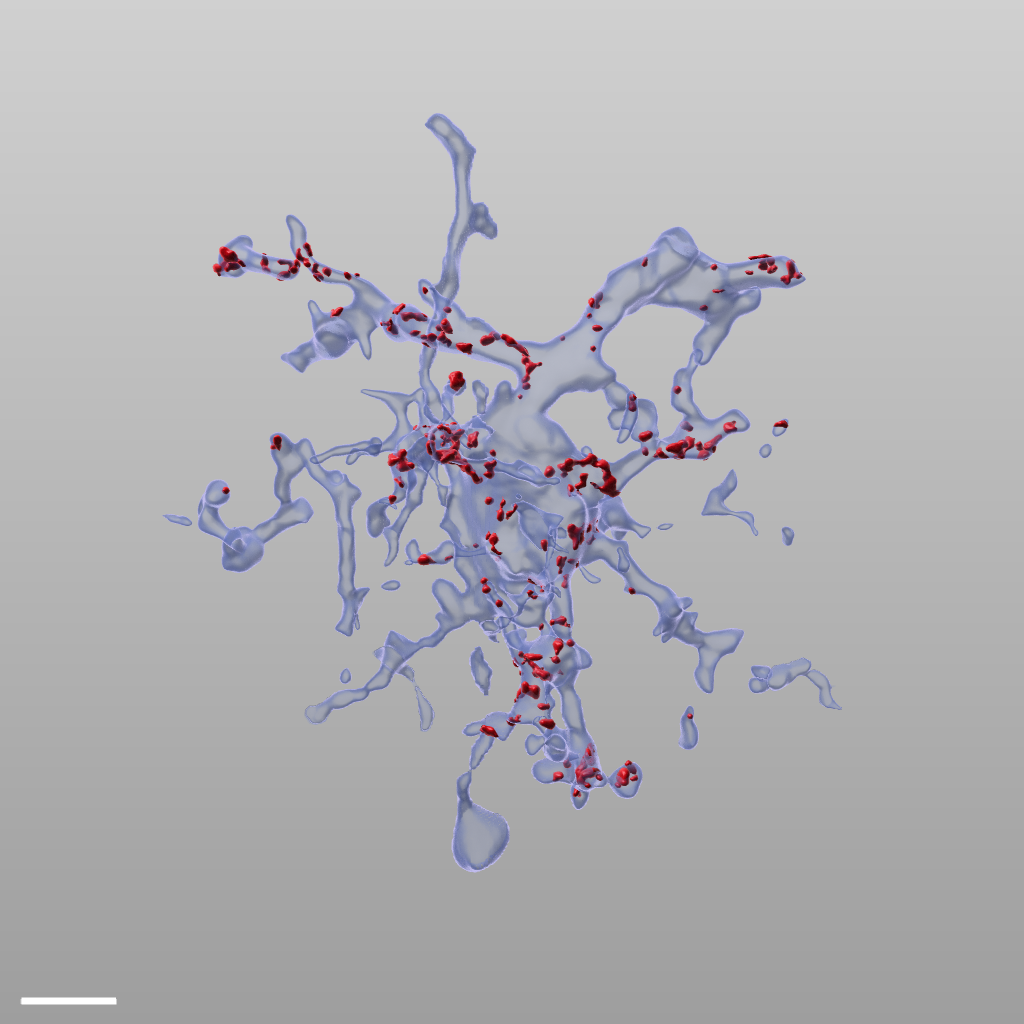

Supplement: Supplementary file 1 [file Data_Sheet_1.zip › raw data 2/FIg5/1231 iba Imported From Huygens_[ii5_POCD7_CA3a_2111_decon_converted_Image_6]_2020-11-24T19-33-12.790.tif]

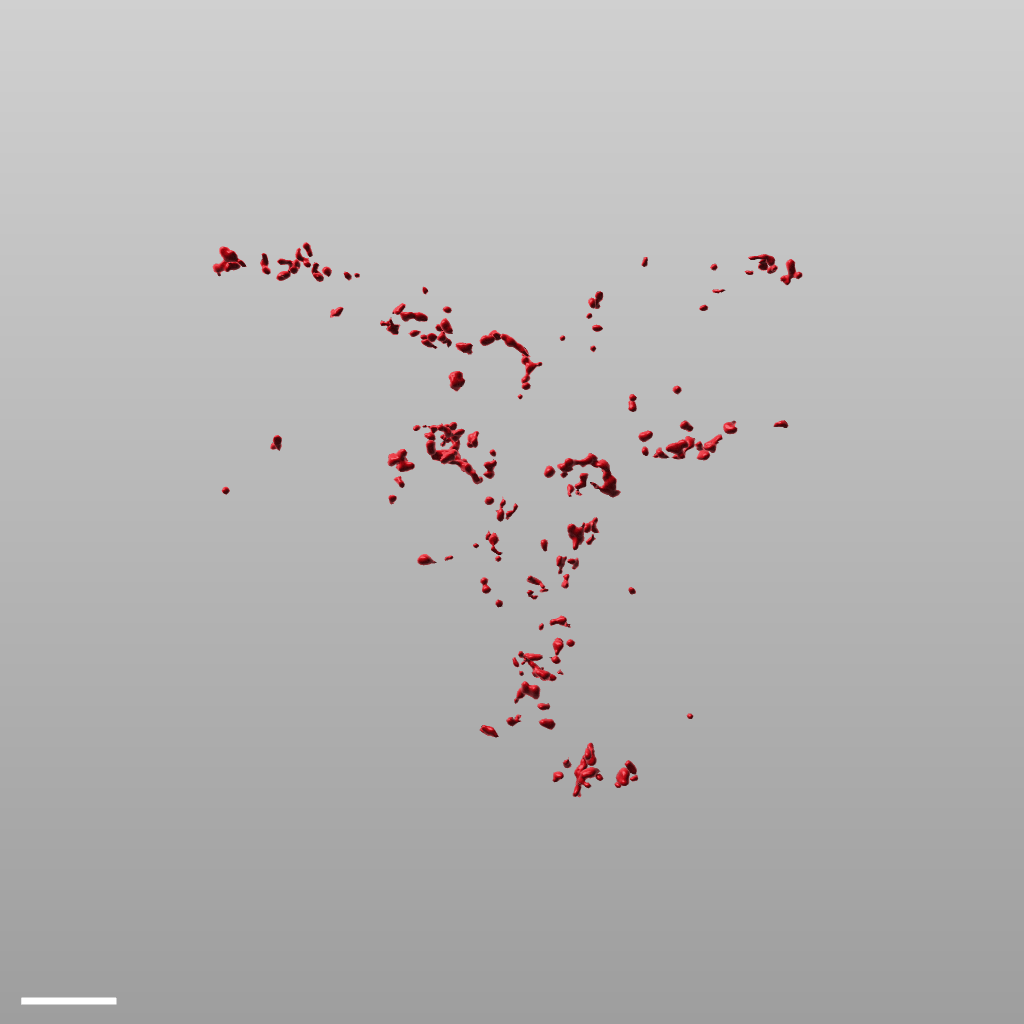

Supplement: Supplementary file 1 [file Data_Sheet_1.zip › raw data 2/FIg5/1231 iba Imported From Huygens_[ii5_POCD7_CA3a_2111_decon_converted_Image_6]_2020-11-24T19-32-51.819.tif]

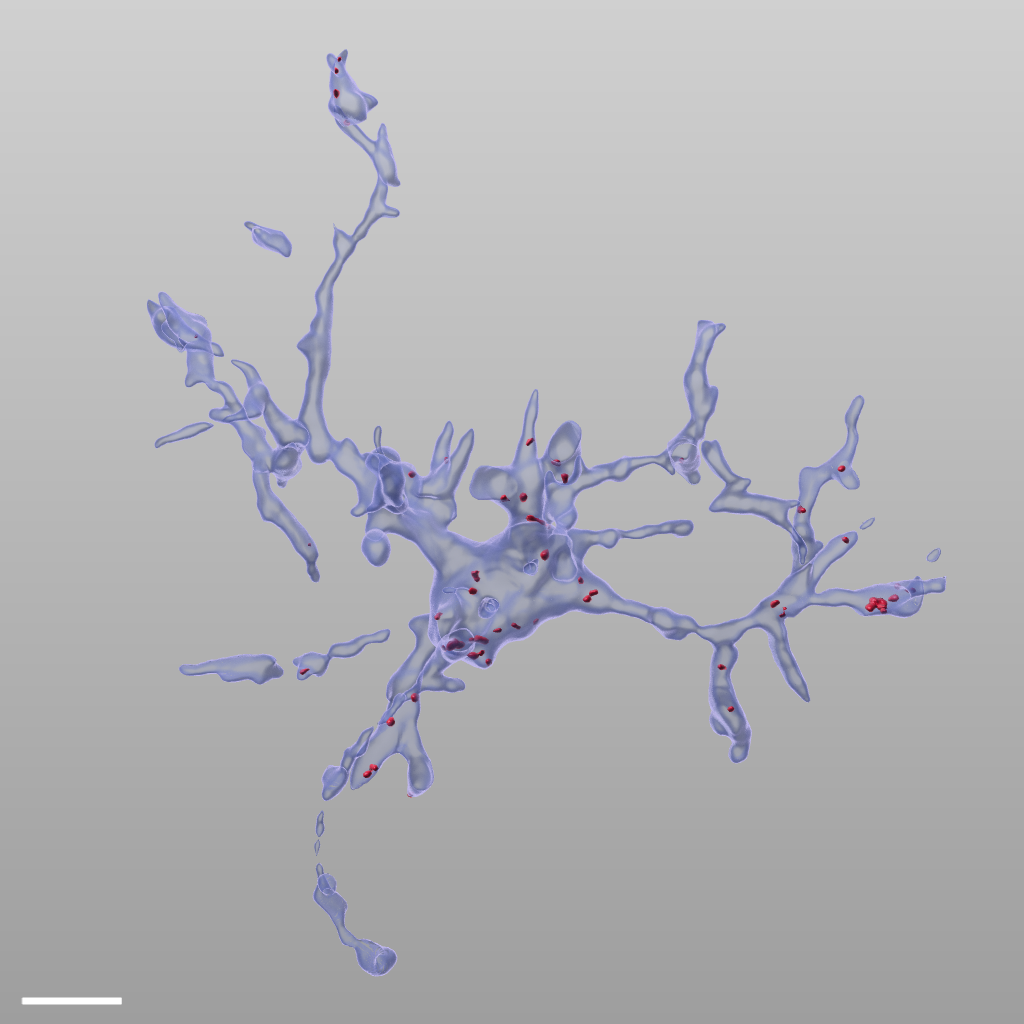

Supplement: Supplementary file 1 [file Data_Sheet_1.zip › raw data 2/FIg5/20200108 IBA Imported From Huygens_[ii6_SIRT2_CA1a_1118_decon_converted_Image_7]_2020-11-24T19-46-12.586.tif]

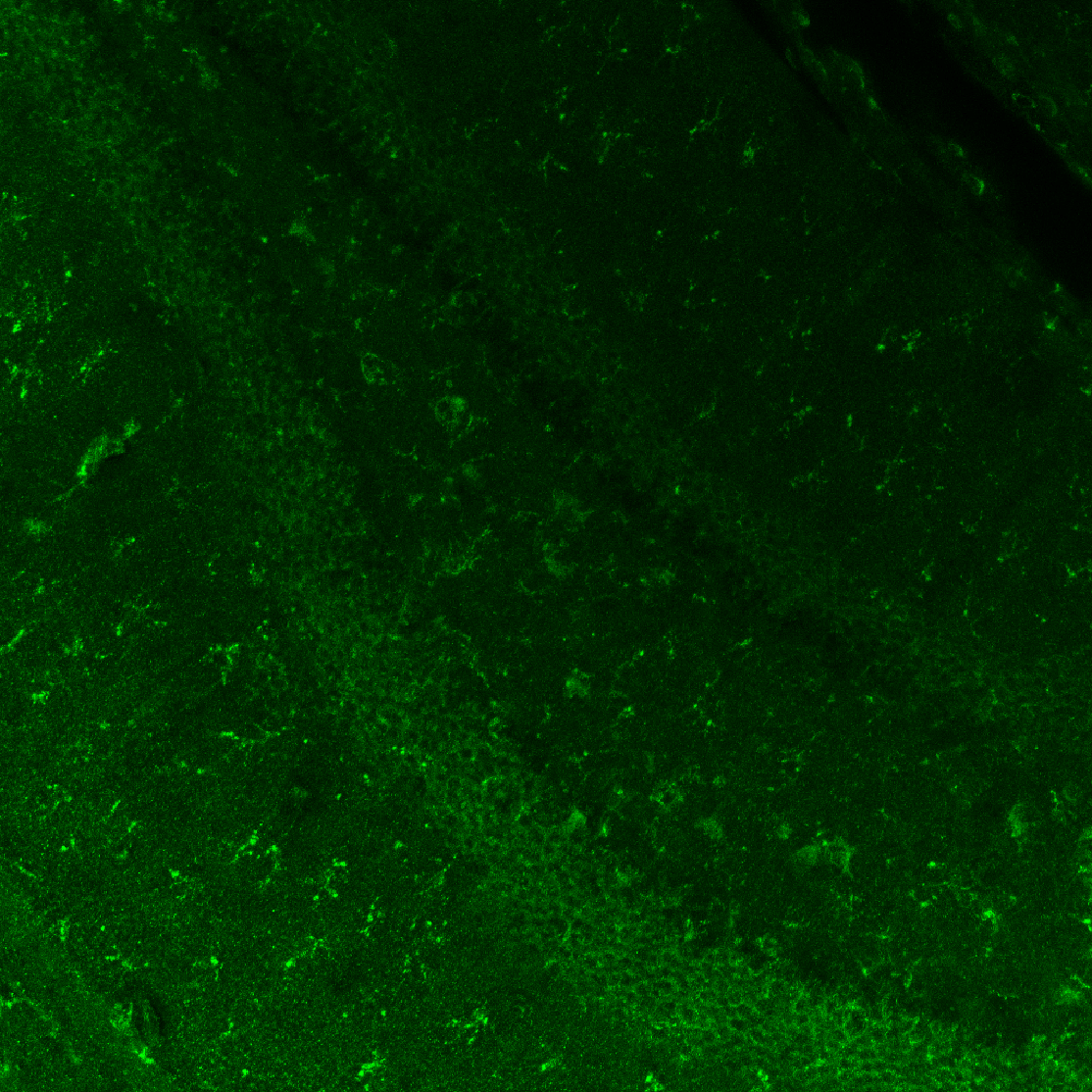

Supplement: Supplementary file 1 [file Data_Sheet_1.zip › raw data 2/FIg5/*20191023 LAMP1001_CTRL_DG_LAMP1 488_2132Snapshot1_ch00.tif]

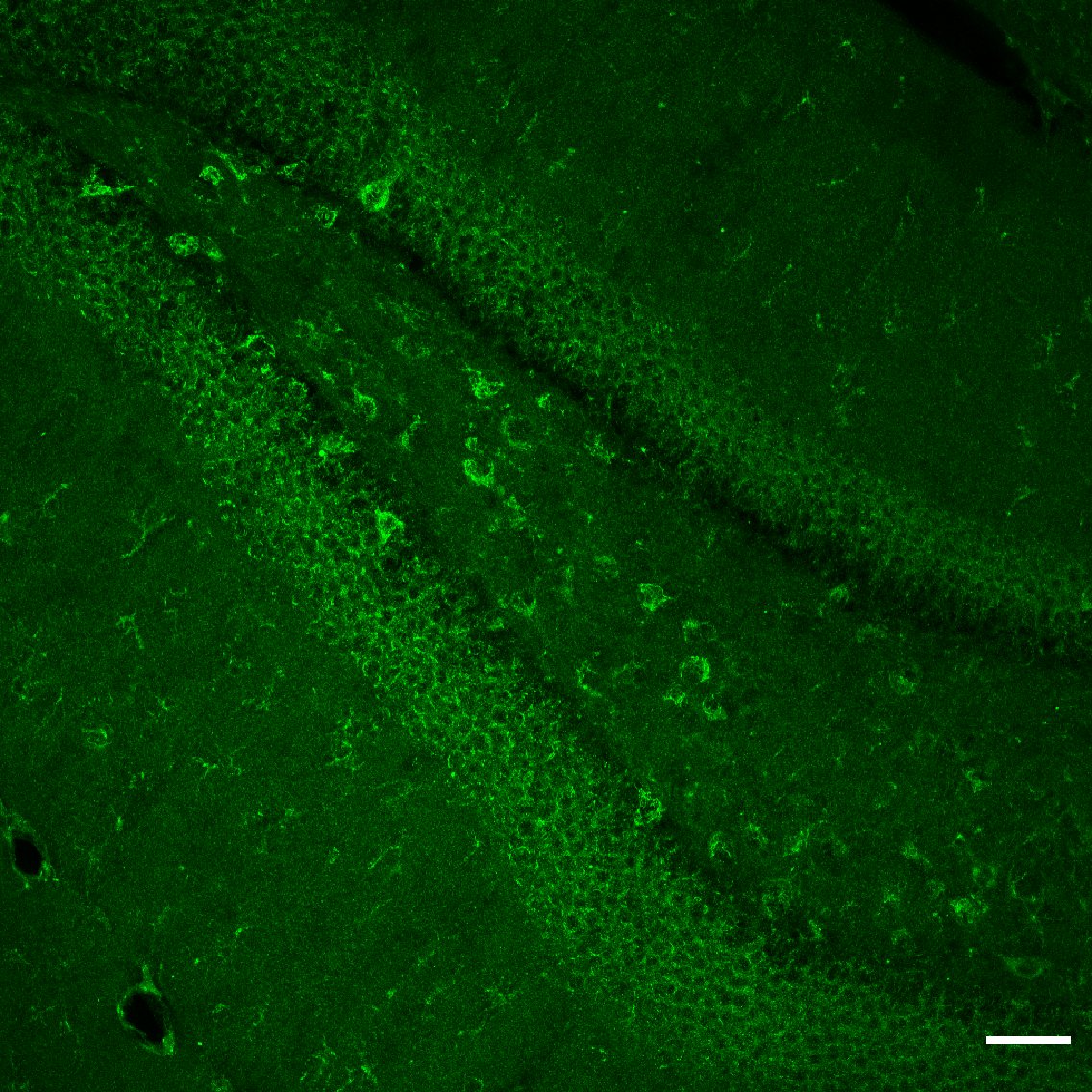

Supplement: Supplementary file 1 [file Data_Sheet_1.zip › raw data 2/FIg5/*20191023 LAMP1001.lif_SIRT_DG_LAMP1 488_2212Snapshot1.jpg]

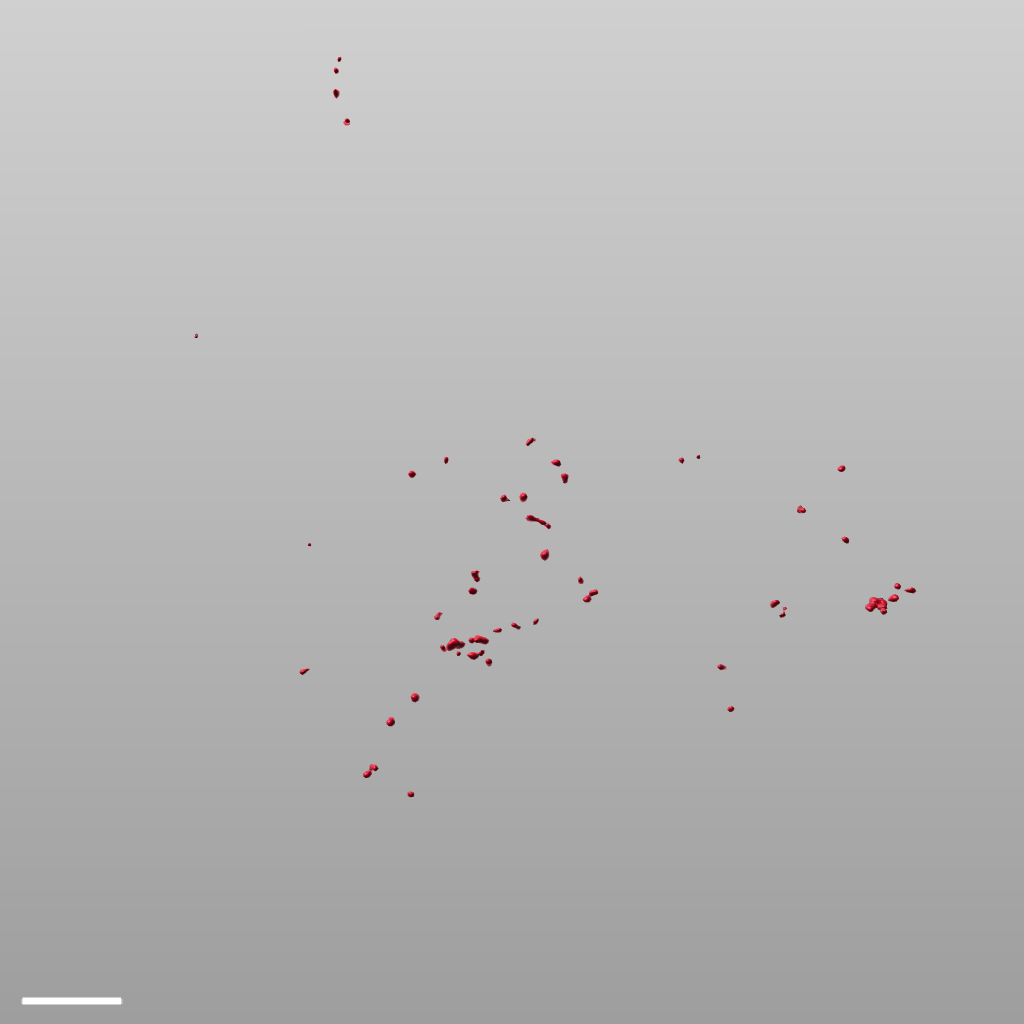

Supplement: Supplementary file 1 [file Data_Sheet_1.zip › raw data 2/FIg5/20200108 IBA Imported From Huygens_[ii6_SIRT2_CA1a_1118_decon_converted_Image_7]_2020-11-24T19-46-03.876.tif]

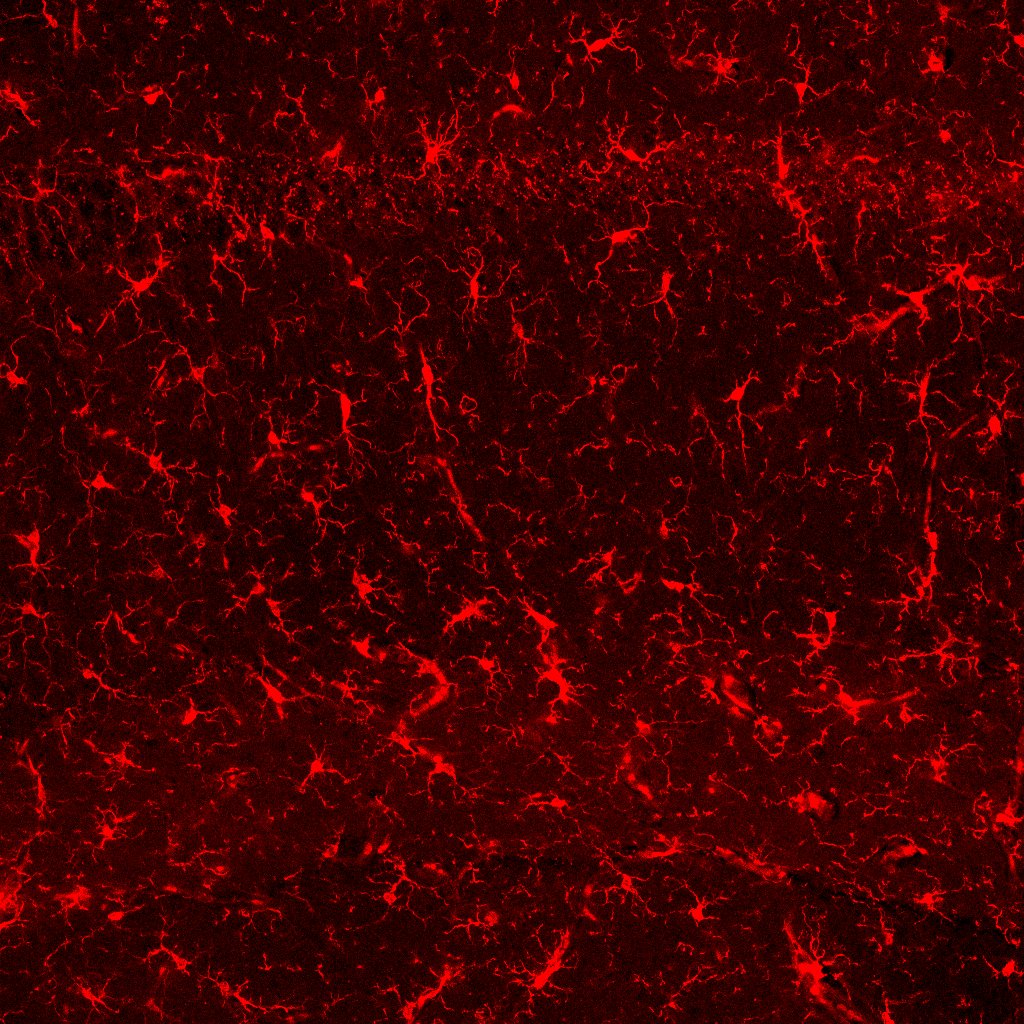

Supplement: Supplementary file 1 [file Data_Sheet_1.zip › raw data 2/Fig3/**d-CTRL_CA1_3112.jpg]

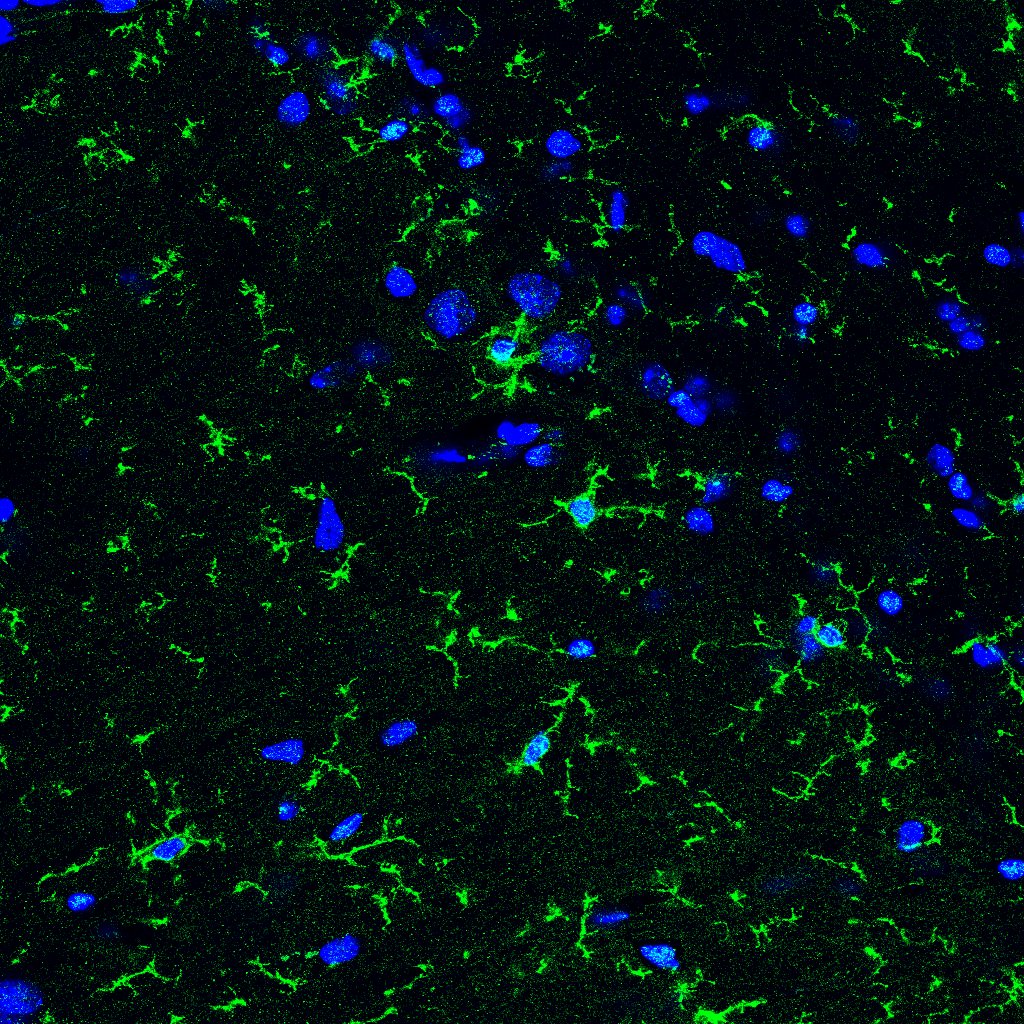

Supplement: Supplementary file 1 [file Data_Sheet_1.zip › raw data 2/Fig3/c-SIRT1+DAPI2.jpg]

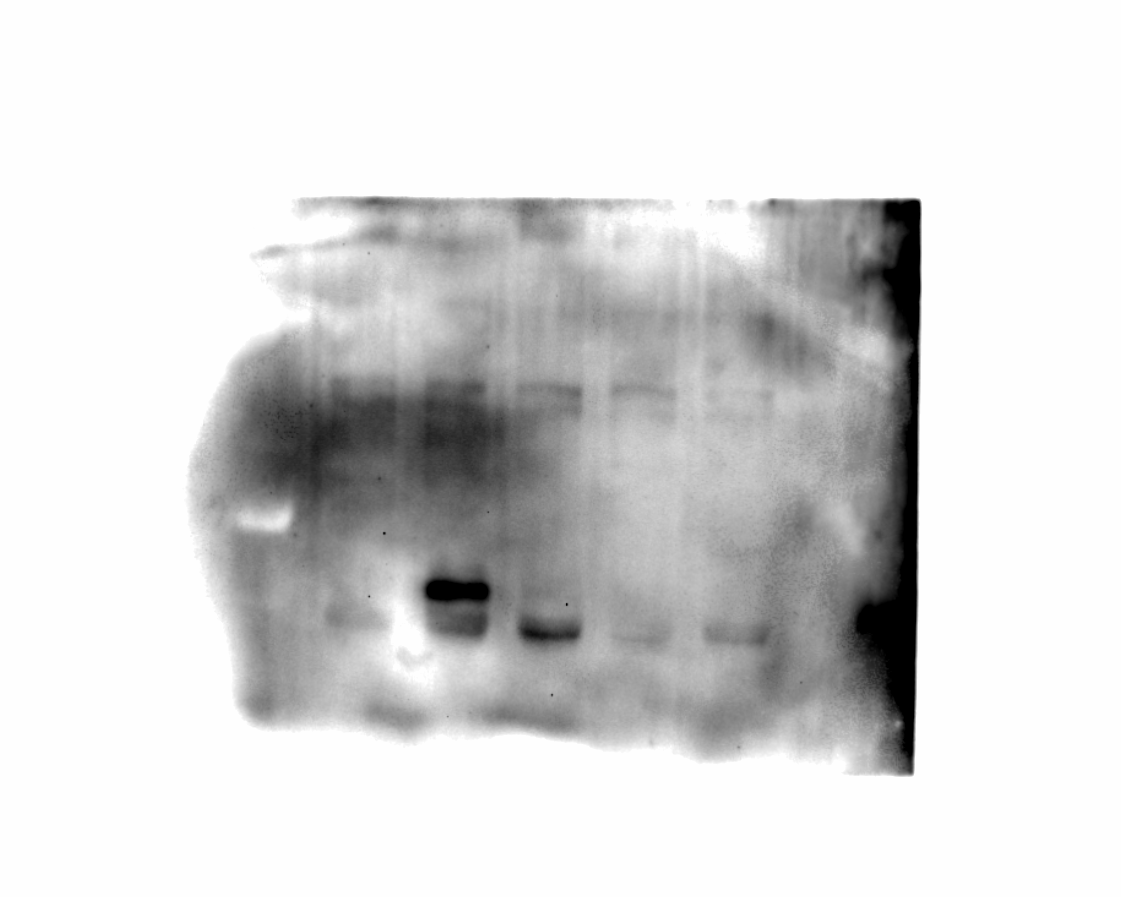

Supplement: Supplementary file 1 [file Data_Sheet_1.zip › raw data 2/Fig3/a-SIRT1 band.tif]

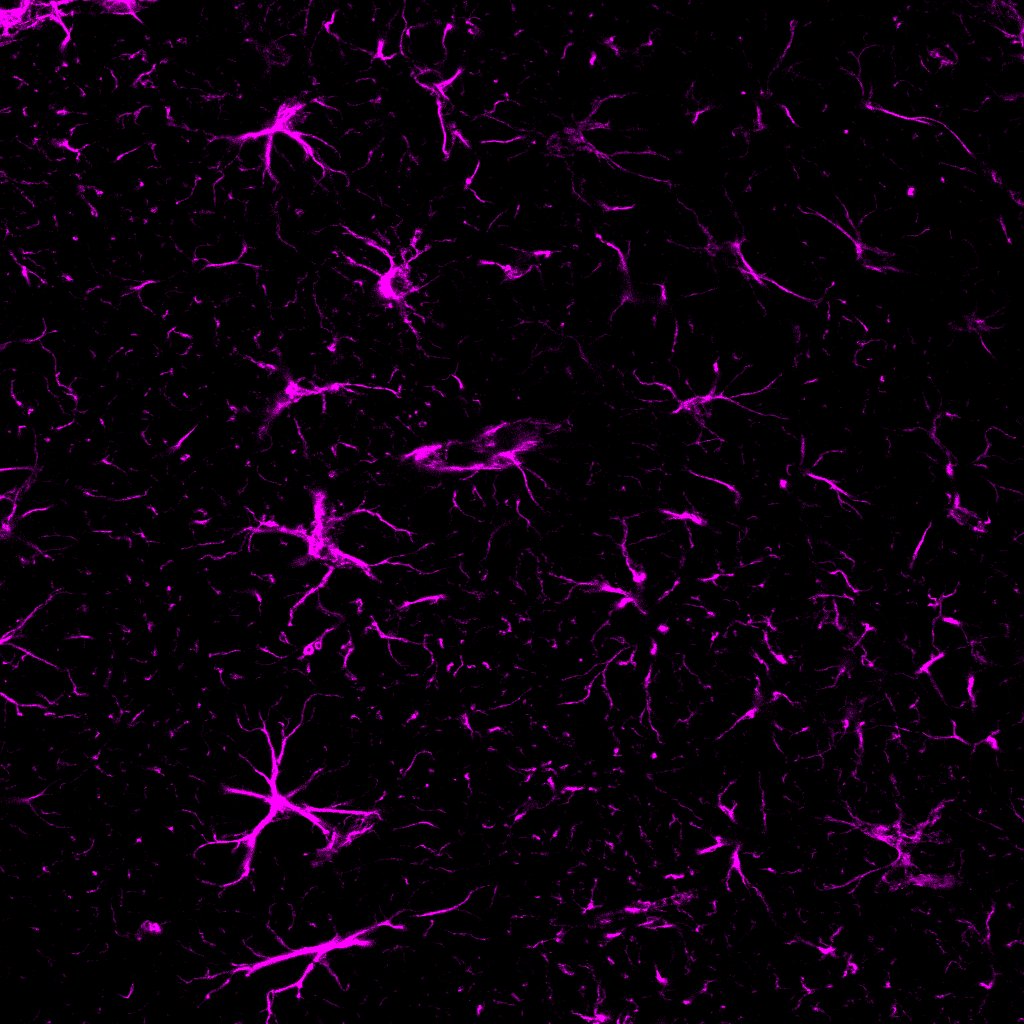

Supplement: Supplementary file 1 [file Data_Sheet_1.zip › raw data 2/Fig3/c-GFAP_gz_SIRT1488_iba568_GFAP647_1151.jpg]

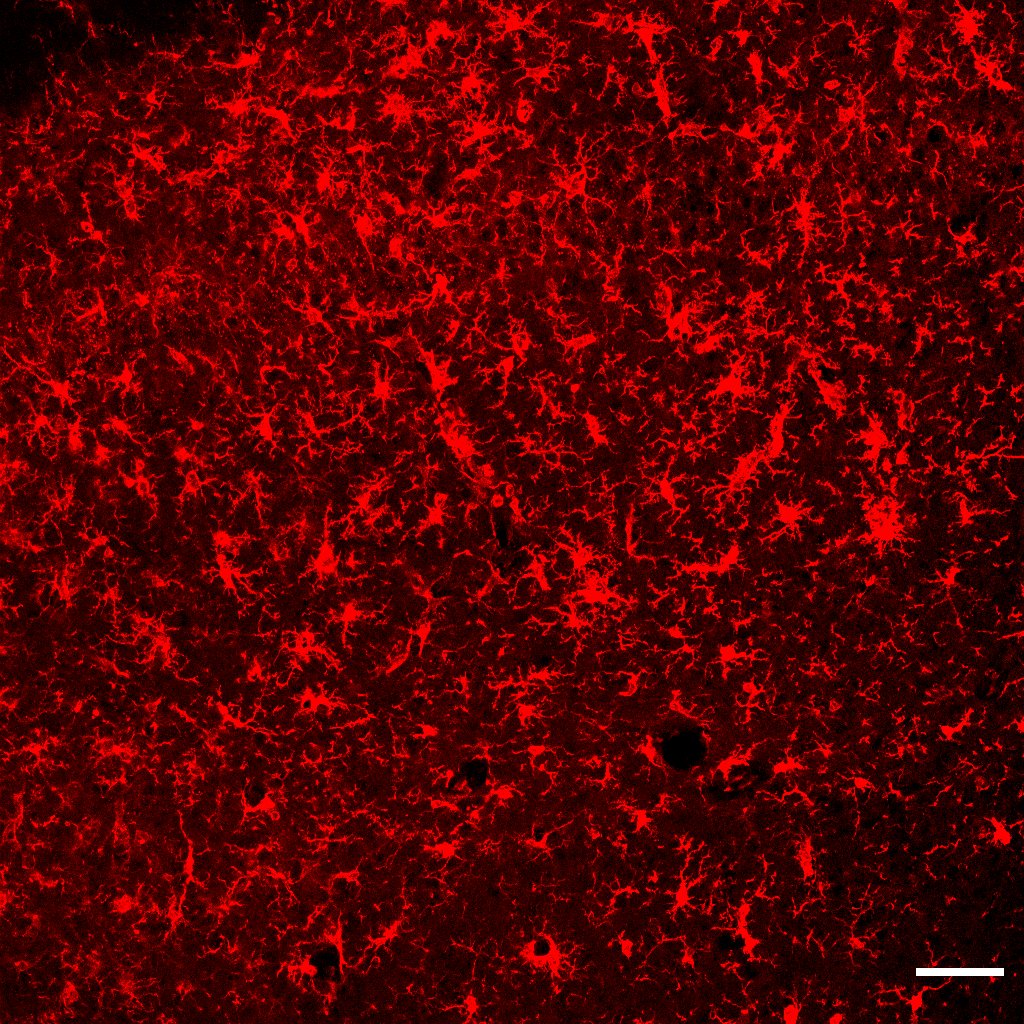

Supplement: Supplementary file 1 [file Data_Sheet_1.zip › raw data 2/Fig3/**d- POCD7_CA1_3131.jpg]

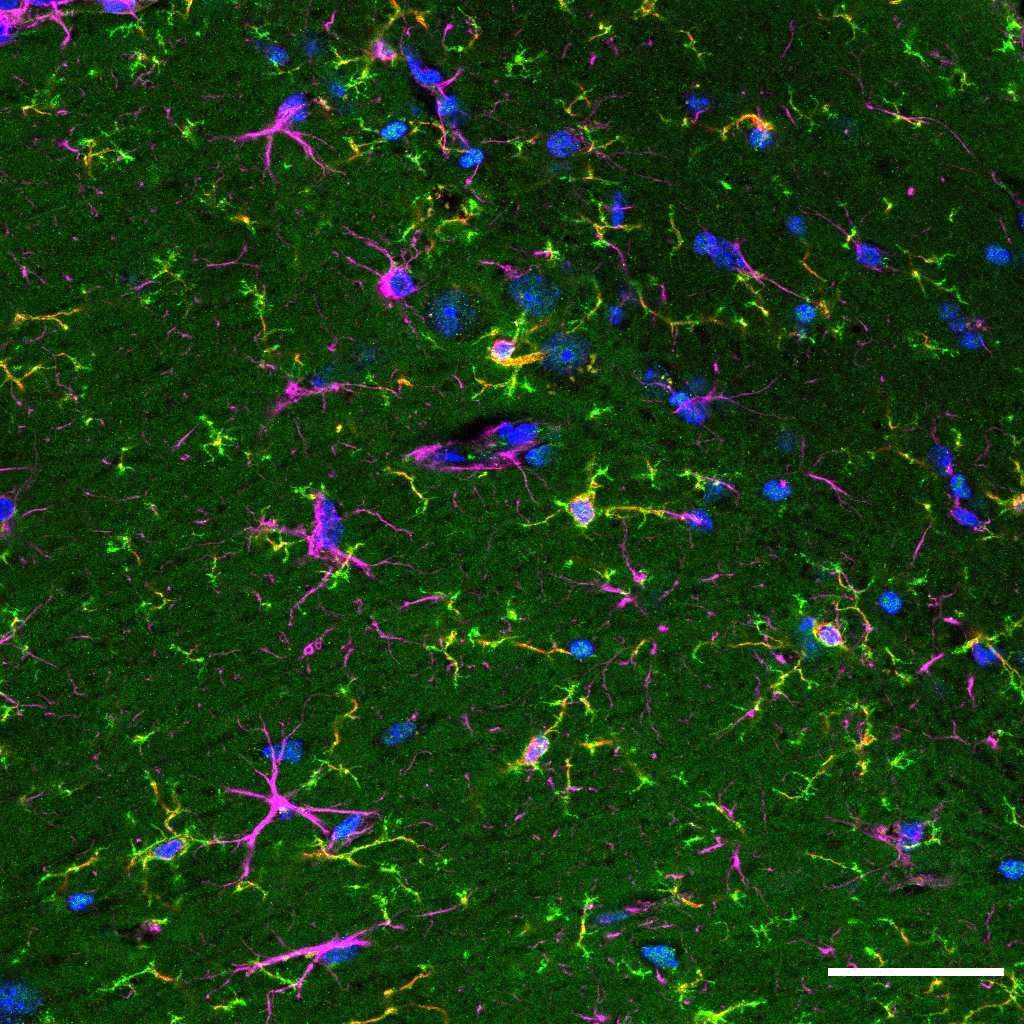

Supplement: Supplementary file 1 [file Data_Sheet_1.zip › raw data 2/Fig3/c-SIRT colabeled.jpg]

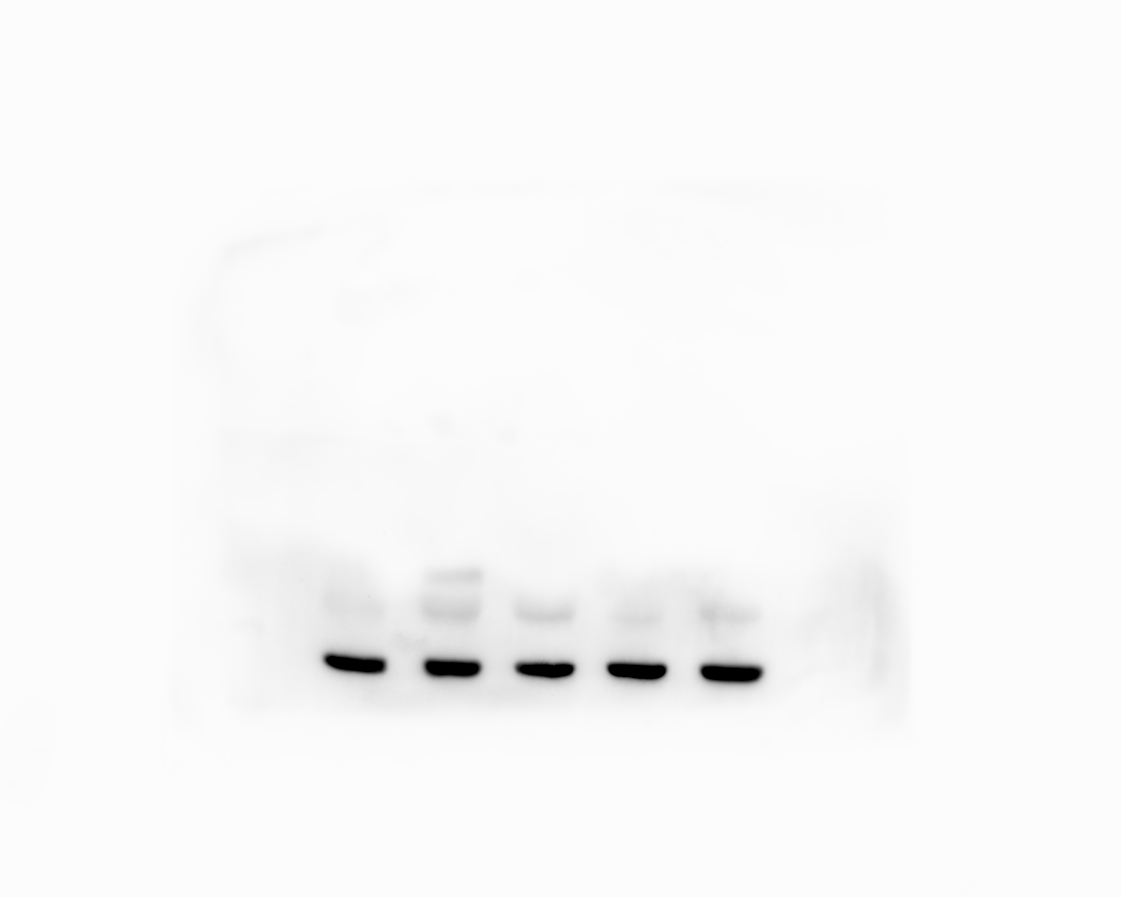

Supplement: Supplementary file 1 [file Data_Sheet_1.zip › raw data 2/Fig3/a-beta-actin.tif]

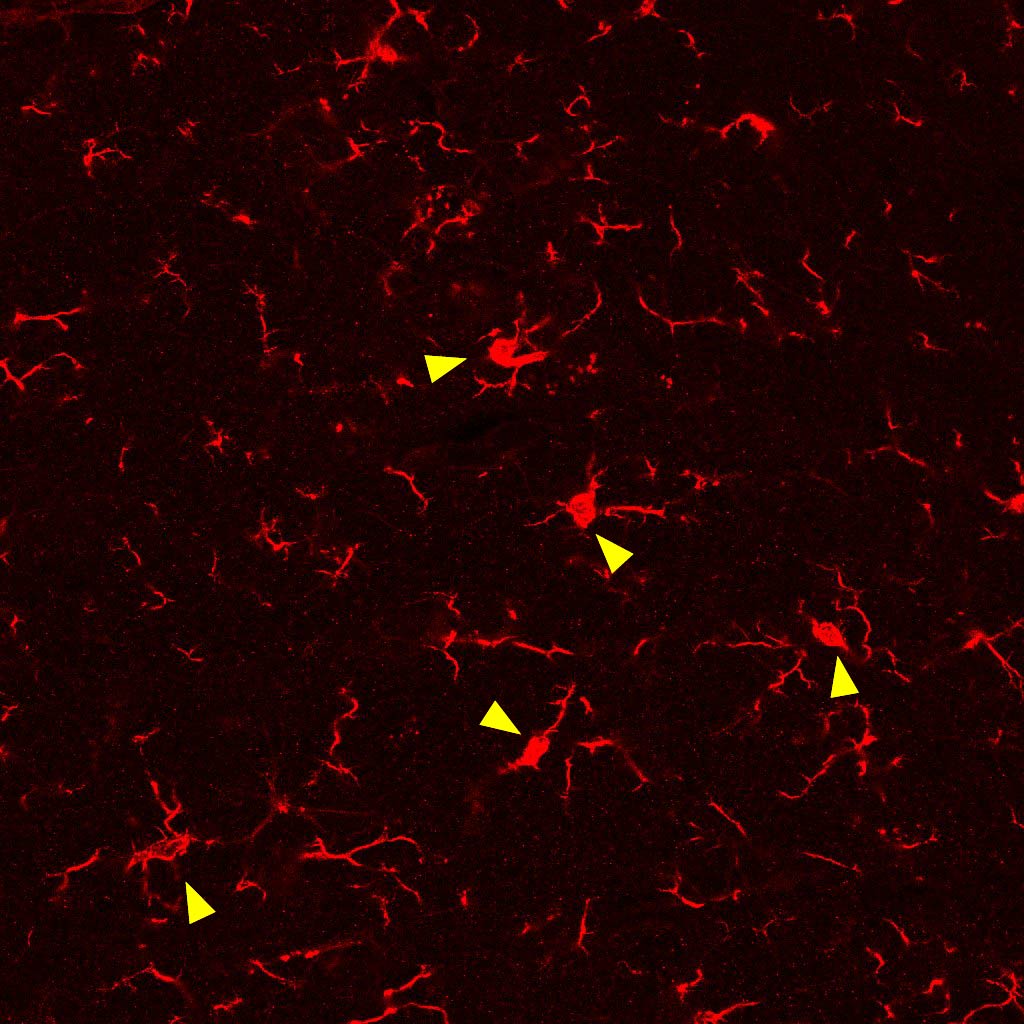

Supplement: Supplementary file 1 [file Data_Sheet_1.zip › raw data 2/Fig3/c-iba_arrow-01.jpg]

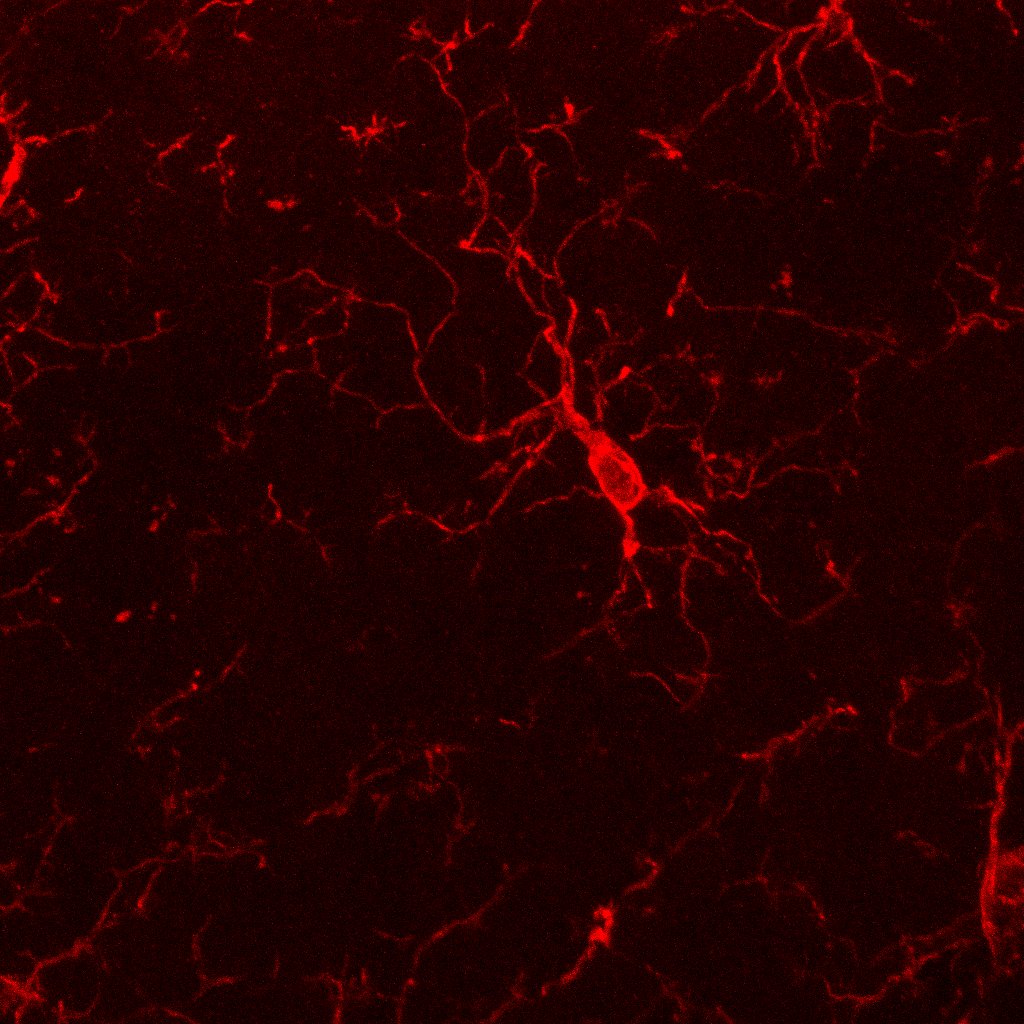

Supplement: Supplementary file 1 [file Data_Sheet_1.zip › raw data 2/Fig4/d-Surgery-SRT1720.jpg]

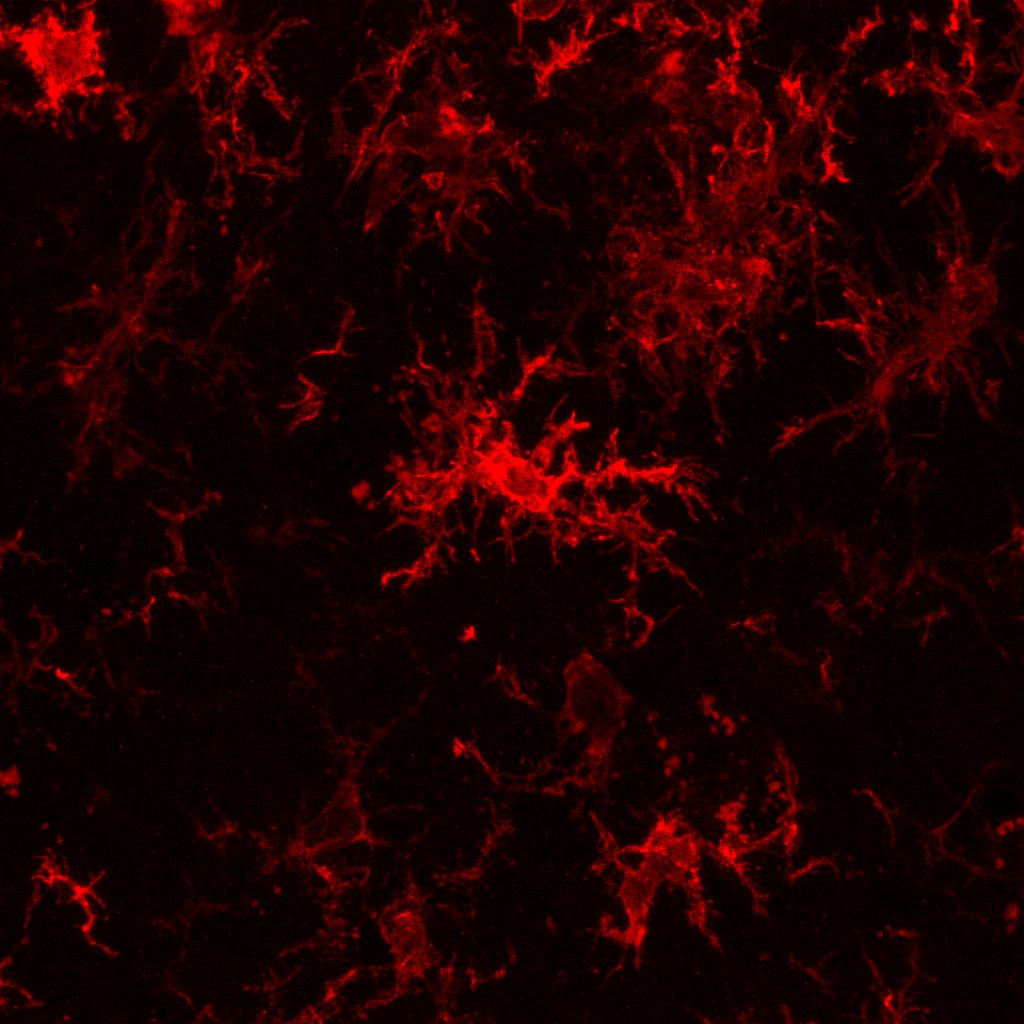

Supplement: Supplementary file 1 [file Data_Sheet_1.zip › raw data 2/Fig4/d-Surgery-vehicle.jpg]

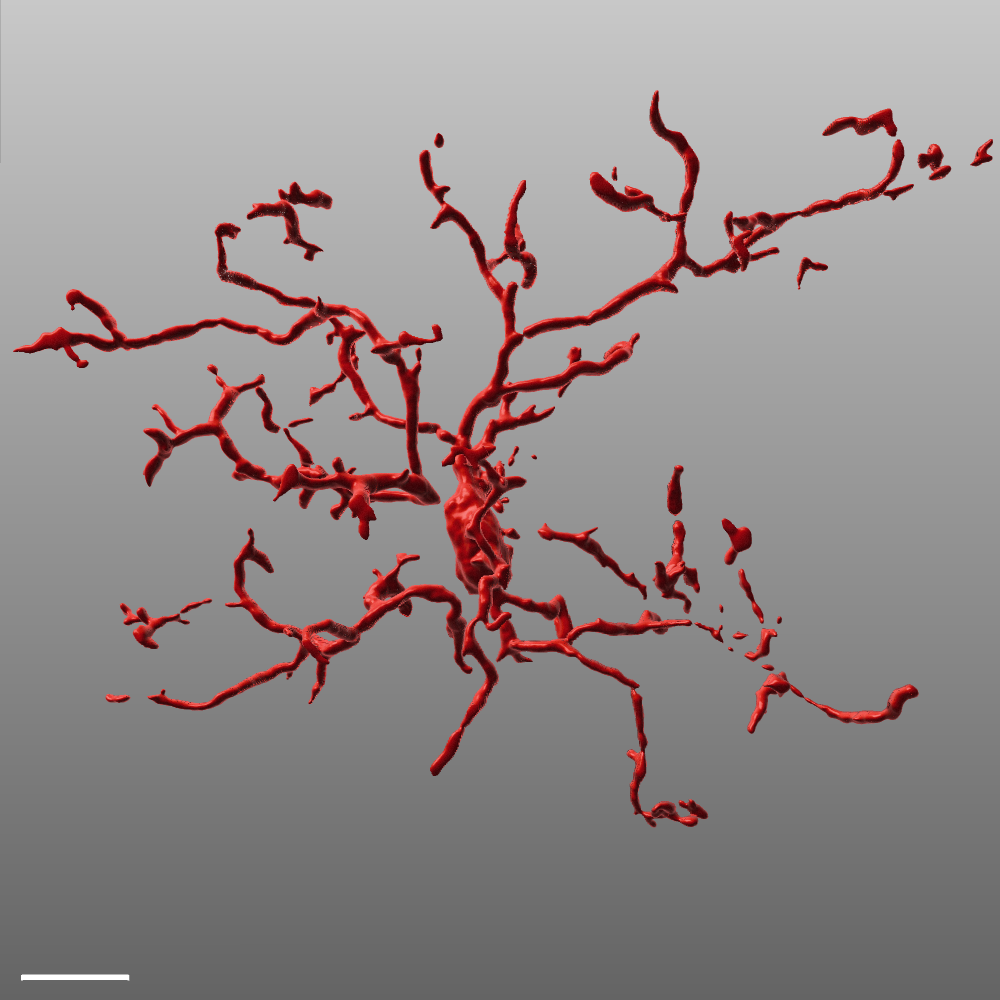

Supplement: Supplementary file 1 [file Data_Sheet_1.zip › raw data 2/Fig4/a-control.tif]

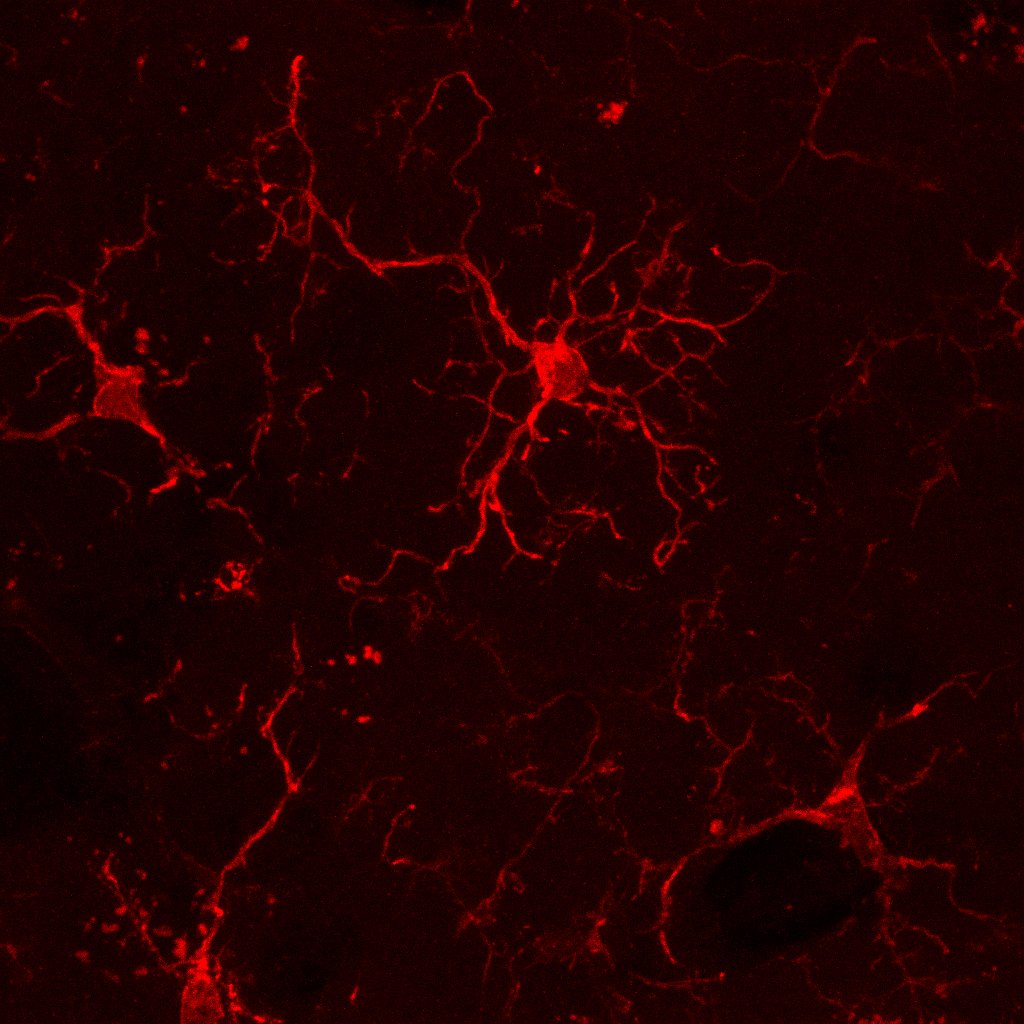

Supplement: Supplementary file 1 [file Data_Sheet_1.zip › raw data 2/Fig4/d-control.jpg]

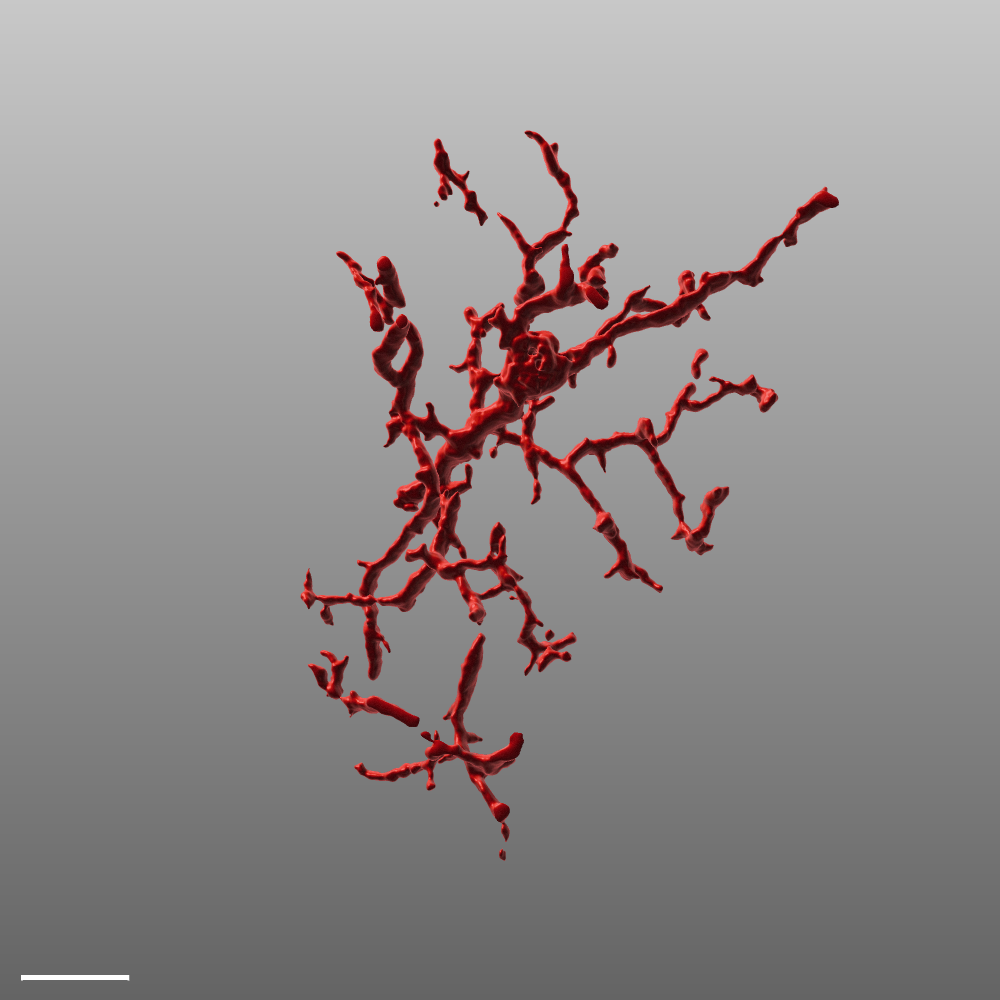

Supplement: Supplementary file 1 [file Data_Sheet_1.zip › raw data 2/Fig4/a-Surgery-SRT1720.tif]

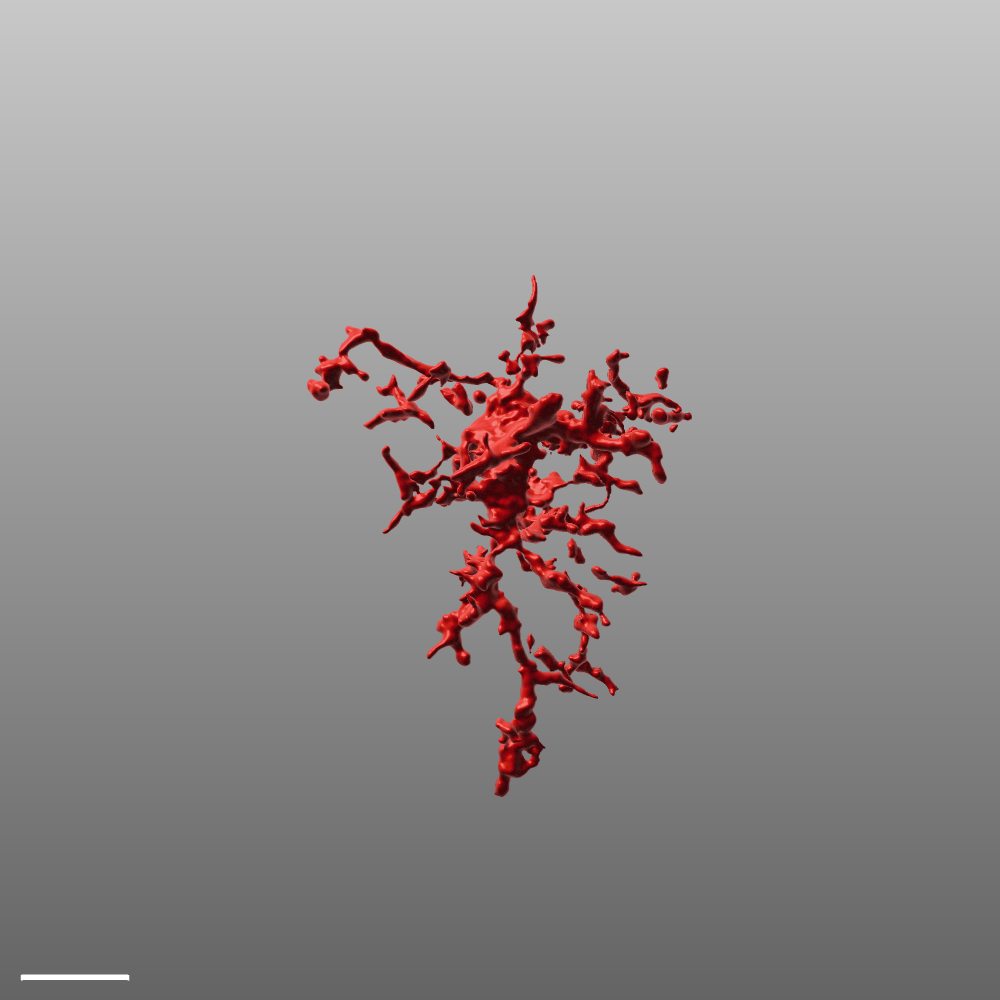

Supplement: Supplementary file 1 [file Data_Sheet_1.zip › raw data 2/Fig4/a-Surgery-vehicle.tif]
